# ATL2 Recruits TRAK1 to Promote Mitochondrial Transport at ER–Mitochondria Contact Sites

**DOI:** 10.1002/advs.76972

**Published:** 2026-07-30

**Authors:** Yiru Cheng, Peiyuan Chai, Xiayuhe Pei, Yiwen Chen, Xiaoshuai Huang, Bei Liu, Yiqian Wu, Junlin Teng, Pengli Zheng, Jianguo Chen

**Affiliations:** ^1^ Key Laboratory of Cell Proliferation and Differentiation of the Ministry of Education, College of Life Sciences Peking University Beijing China; ^2^ Center For Life Sciences Academy for Advanced Interdisciplinary Studies Peking University Beijing China; ^3^ Beijing Advanced Center of Cellular Homeostasis and Aging‐Related Diseases, Institute of Advanced Clinical Medicine Peking University Beijing China; ^4^ National Biomedical Imaging Center, College of Future Technology Peking University Beijing China; ^5^ Center for Quantitative Biology Academy for Advanced Interdisciplinary Studies Peking University Beijing China

**Keywords:** ATL2, ER–mitochondria contact sites, hypoxia, mitochondrial transport, TRAK1

## Abstract

Mitochondrial transport and distribution are crucial for cellular homeostasis, yet whether and how they are regulated by endoplasmic reticulum (ER)–mitochondria contact sites remains unclear. Here, we demonstrate that the ER protein atlastin‐2 (ATL2) orchestrates mitochondrial transport and distribution by promoting assembly of the transport machinery at ER–mitochondria contact sites. Mechanistically, ATL2 recruits the adaptor trafficking kinesin‐binding protein 1 (TRAK1) to the ER membrane, strengthening the interaction of TRAK1 with the mitochondrial transport adaptor MIRO1 to promote anterograde mitochondrial transport. Loss of ATL2 disrupts this process, leading to perinuclear mitochondrial clustering. We further find that ATL2 stabilizes ER–mitochondria contact sites by interacting with MFN2, providing a platform for mitochondrial transport complex assembly. Moreover, in hypoxia, ATL2 is ubiquitinated at lysine 567 by the E3 ligase SYVN1, leading to its degradation and a resulting defect in mitochondrial distribution. Our findings elucidate a novel ER‐mediated mechanism for mitochondrial transport.

## Abstract 2

ER–mitochondria contacts orchestrate mitochondrial transport via ATL2. The ER protein ATL2 promotes the TRAK1–MIRO1 interaction by recruiting TRAK1 to the ER and by interacting with mitochondrial MFN2 at contact sites. This facilitates assembly of the transport adaptor complex, which in turn recruits the motor protein KIF5B to drive anterograde mitochondrial transport.

## 1 Introduction

Mitochondria are highly dynamic organelles that not only produce cellular energy but also require precise intracellular transport and distribution to support local metabolic demands and maintain overall cellular fitness. Defects in these processes are now widely implicated in the pathogenesis of major human diseases [1, 2, 3]. Mitochondria undergo bidirectional transport along microtubules. The kinesin‐1 motor primarily mediates their anterograde transport toward the microtubule plus‐end, whereas retrograde movement toward the minus‐end is driven by the cytoplasmic dynein–dynactin motor complex [4, 5]. Kinesin‐1 is a heterotetramer composed of two heavy and two light chains, in which the heavy chain N‐terminus binds microtubules, and its C‐terminus interacts with cargo or light chains [6, 7]. Mammals express three heavy chain isoforms that exhibit distinct expression patterns: KIF5B is widespread, whereas KIF5A and KIF5C are largely neuron‐specific [8]. A functionally conserved adaptor complex forms the critical link between these motors and mitochondria. The central components are the trafficking kinesin‐binding proteins (TRAKs), which recruit these motors to mitochondria via the membrane‐anchored GTPase MIRO [5, 9, 10, 11]. The TRAK–MIRO adaptor complex in mammals comprises paralogous pairs, TRAK1/2 and MIRO1/2 [12, 13, 14]. Beyond this core complex, other adaptor proteins, including FEZ1, syntabulin, RanBP2, ARMCX1, ARMCX3, and metaxin, also contribute to mitochondrial motility [15, 16, 17]. A central remaining question is which adaptor combinations are recruited for mitochondrial transport under specific conditions, and how upstream signals regulate this recruitment.

The ER and mitochondria form specialized membrane contact sites, also known as mitochondria‐associated ER membranes (MAMs), which are platforms for lipid exchange, calcium signaling, and regulation of mitochondrial dynamics [18, 19]. Many proteins have been identified as membrane tethers at ER–mitochondria contact sites [20]. Among these, the mitochondrial fusion protein mitofusin 2 (MFN2) forms interorganellar tethers through homotypic or heterotypic interactions with MFN1or MFN2 [21, 22]. Given the persisting uncertainty regarding the ER localization of MFN2, it is plausible that additional, yet unidentified, ER‐resident proteins cooperate with MFN2 to facilitate this interorganellar tethering. Beyond these established roles, ER–mitochondria contact sites also regulate mitochondrial transport [23], yet the precise mechanisms underlying this coordination remain unclear.

The dynamin‐like GTPase atlastin (ATL) is the master regulator of ER membrane fusion [24, 25]. Mammals express three paralogs with distinct expression patterns: brain‐enriched ATL1 and the ubiquitously expressed ATL2 and ATL3 [26]. Beyond their canonical ER fusion activity, ATL proteins also exhibit membrane‐tethering capabilities [27, 28]. The physiological significance of the ATL family is demonstrated by its disease associations, ranging from genetic mutations of ATL1 and ATL3 in hereditary neurodegenerative diseases to elevated ATL2 expression in models of Alzheimer's disease [29, 30, 31]. Furthermore, embryonic lethality and cerebellar defects in *ATL2*‐knockout mice demonstrate its critical role in development [32]. Although mitochondrial transport deficits in ATL1‐mutant patient‐derived cells indicate a role for ATLs in regulating mitochondrial motility [33], the mechanistic basis of this involvement remains elusive.

In this study, we demonstrate that ATL2 regulates mitochondrial transport by recruiting TRAK1 to promote assembly of the TRAK1–MIRO1 complex. Furthermore, we show that ATL2–MFN2 interactions reinforce organelle tethering, creating a structural platform for mitochondrial transport. Finally, we reveal that hypoxia disrupts this process by promoting ubiquitin‐dependent degradation of ATL2. Our work thus provides a mechanistic framework for understanding how ER–mitochondria contact sites govern mitochondrial transport.

## 2 Results

### 2.1 ATL2 Promotes Mitochondrial Anterograde Transport and Proper Distribution

To investigate whether ATL2 regulates mitochondrial transport, we first generated *ATL2* knockout (KO) in COS‐7 cells, which do not endogenously express ATL1 [34]. In *ATL2* KO cells, mitochondria were prominently clustered around the nucleus, whereas in *ATL3* KO cells, mitochondrial distribution was dispersed and similar to that in wild‐type (WT) cells (Figure 1A,B). To quantitatively assess this phenotype, we used the mitochondrial mean distribution radius (MDR) assay as previously described [35], which reflects the average distance of mitochondria from the nuclear envelope to the plasma membrane (higher MDR values indicate more peripheral localization). Consistent with the morphological observations, *ATL2* KO cells, but not *ATL3* KO cells, showed a significantly smaller MDR than WT cells (Figure 1C).

**FIGURE 1:**
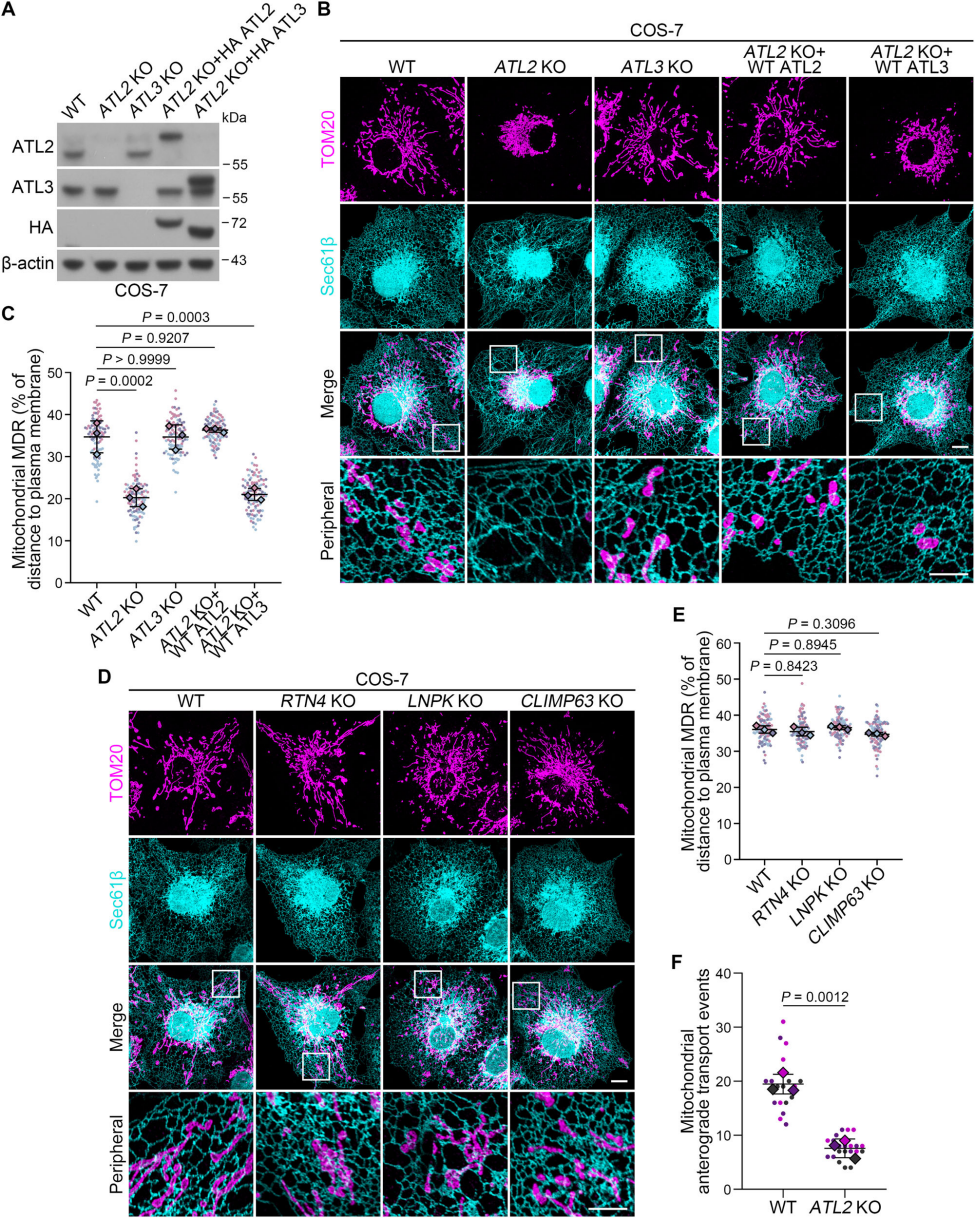
ATL2 promotes mitochondrial anterograde transport and appropriate distribution. (A) Immunoblot analysis of ATL2 and ATL3 in wild‐type (WT), *ATL2* knockout (KO), *ATL3* KO, and *ATL2* KO COS‐7 cells stably reconstituted with ATL2 or ATL3. (B) Representative images of cells, genotypes as shown in (A), stably expressing 3×mEmerald‐Sec61β (cyan, ER marker) and labelled with TOM20 (magenta, mitochondrial marker). Peripheral regions are enlarged at the bottom. Scale bars, 10 µm; 5 µm (inset). (C) Mitochondrial mean distribution radius (MDR) in cells as shown in (B). *n* = 102, 104, 103, 104, and 103 cells from three biological replicates. Biological replicates are denoted by color, with individual MDR depicted as smaller points. Data are presented as mean ± s.d. across biological replicates. (D) Representative images of WT, knockout of *RTN4*, *LNPK*, and *CLIMP63* in COS‐7 cells stably expressing 3×mEmerald‐Sec61β (cyan) and labelled with TOM20 (magenta). Peripheral regions are enlarged at the bottom. Scale bars, 10 µm; 5 µm (inset). (E) Mitochondrial MDR in cells as in (D). *n* = 104, 102, 104, and 105 cells from three biological replicates. Biological replicates are denoted by color, with individual MDR values depicted as smaller points. Data are presented as mean ± s.d. across biological replicates. (F) Mitochondrial anterograde transport events over 10 min in cells as shown in Figure S1I. *n* = 19 and 21 cells from three biological replicates. Biological replicates are denoted by color, with individual transport events depicted as smaller points. Data are presented as mean ± s.d. across biological replicates. Statistical analyses were performed using ordinary one‐way analysis of variance (ANOVA) followed by Tukey's multiple comparisons test (C and E) and two‐tailed unpaired *t*‐tests (F).

ATL2 deletion leads to aberrant ER morphology, characterized by reduced tubule branching. Re‐expression of ATL3 rescued this ER structural defect, owing to functional redundancy of ATL paralogs during ER morphogenesis [36]. Consistent with those findings, re‐expression of either ATL2 or ATL3 in *ATL2* KO cells restored normal ER morphology. However, only re‐expression of ATL2 reversed the perinuclear mitochondrial clustering phenotype, whereas ATL3 re‐expression failed to rescue mitochondrial distribution despite correcting ER structure (Figure 1B,C). To extend these findings to another cell line, we analyzed mitochondrial distribution in HeLa cells and confirmed the perinuclear clustering of mitochondria in ATL2 KO HeLa cells (Figure S1A–C).

To further exclude the possibility that the mitochondrial distribution defect in *ATL2* KO cells is an indirect consequence of altered ER morphology, we generated KO cell lines targeting additional ER‐shaping proteins (RTN4, lunapark, and CLIMP63) in COS‐7 cells (Figure S1D). As previously reported [37, 38, 39], deletion of these proteins caused distinct ER morphological abnormalities compared to WT cells; however, none affected mitochondrial distribution (Figure 1D,E). These results indicate that, among proteins involved in ER morphology, ATL2 specifically regulates mitochondrial distribution, independent of its established role in ER shaping.

Immunoblotting for the mitochondrial matrix protein HSP60 and the outer membrane protein TOM20 showed comparable expression levels in WT and *ATL2* KO COS‐7 cells (Figure S1E,F). Similarly, immunoblotting analysis in HeLa cells showed that the levels of HSP60, TOM20, and the inner membrane protein TIM50 were unaffected by ATL2 deletion (Figure S1G,H). These data demonstrate that the perinuclear mitochondrial clustering phenotype does not arise from reduced mitochondrial mass.

To test whether ATL2 deletion alters mitochondrial transport, we labeled mitochondria to visualize and track their dynamics in WT and *ATL2* KO COS‐7 cells. We observed significantly fewer mitochondrial anterograde transport events upon ATL2 deletion, with no significant effect on retrograde mitochondrial transport, resulting in perinuclear mitochondrial clustering (Figure 1F and Figure S1I,J). Therefore, the ER‐resident protein ATL2 emerges as a regulator of mitochondrial distribution, likely by modulating anterograde mitochondrial transport.

To investigate whether ATL2 loss alters mitochondrial morphology, we analysed WT and *ATL2* KO HeLa cells by transmission electron microscopy (TEM) and quantified mitochondrial length and aspect ratio. The loss of ATL2 did not affect either parameter (Figure S1K,L), indicating that the mitochondrial trafficking and distribution defects caused by ATL2 deficiency are independent of mitochondrial morphology.

### 2.2 ATL2 Promotes Mitochondrial Transport and Distribution in a KIF5B‐Dependent Manner

We next sought to uncover the upstream mechanism by which ATL2 facilitates anterograde mitochondrial transport. Since microtubule stability serves as the fundamental track supporting mitochondrial trafficking, a key determinant of mitochondrial transport [40], we first examined whether loss of ATL2 impairs microtubule integrity. Cells were treated with nocodazole, a microtubule‐destabilizing agent, and total α‐tubulin fluorescence intensity was quantified. No significant difference in tubulin signals was observed between WT and *ATL2* KO COS‐7 cells following drug exposure, ruling out microtubule destabilization as the cause of defective mitochondrial distribution (Figure S2A). We then applied proximity ligation assay (PLA) to visualize physical associations between mitochondria and microtubules. The PLA signal marking tubulin‐bound mitochondria was markedly weaker in *ATL2* KO HeLa cells relative to WT controls (Figure S2B), indicating that ATL2 depletion disrupts the recruitment of mitochondria to microtubule tracks.

KIF5B is a ubiquitously expressed kinesin‐1 motor essential for anterograde mitochondrial transport along microtubules and is required for peripheral mitochondrial distribution. Loss of KIF5B function causes perinuclear clustering of mitochondria [41]. We identified an interaction between ATL2 and KIF5B using co‐immunoprecipitation and glutathione S‐transferase (GST) pull‐down assays (Figure 2A–C and Figure S2C). Domain mapping revealed that ATL2 binds the KIF5B tail domain (residues 745–963) (Figure S2D,E), which coincides with the known binding site for TRAK adaptor proteins [42].

**FIGURE 2:**
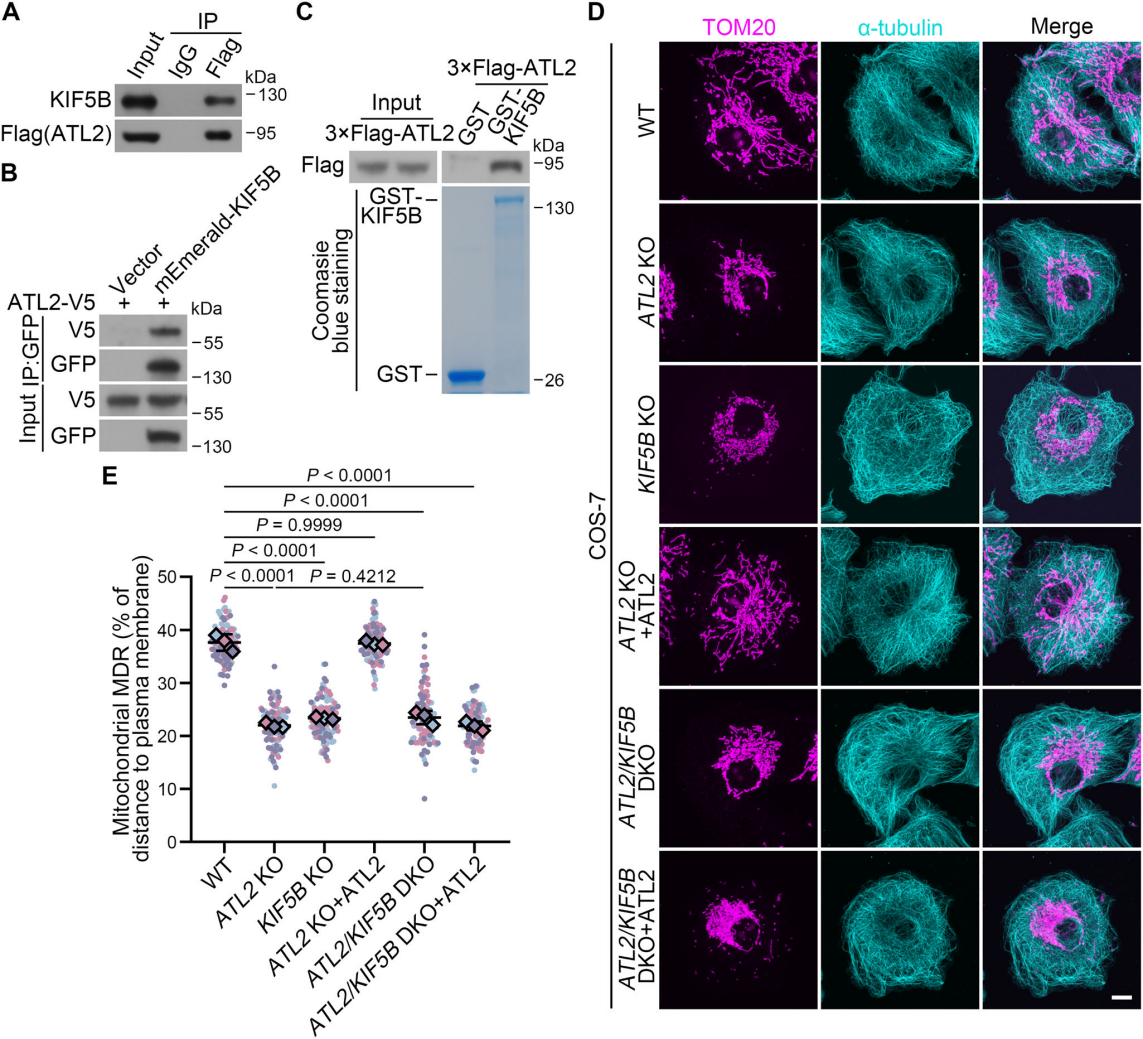
ATL2 promotes mitochondrial transport and distribution in a KIF5B‐dependent manner. (A) Lysates from *ATL2* knockout (KO) HeLa cells stably reconstituted with 3×Flag‐ATL2 were subjected to immunoprecipitation (IP) using control IgG or anti‐Flag antibody‐conjugated beads. Immunoprecipitates were analysed by immunoblotting using antibodies against Flag and KIF5B. (B) HEK293T cells co‐transfected with ATL2‐V5, and either mEmerald‐KIF5B or the mEmerald‐C1 vector were subjected to immunoprecipitation using anti‐GFP nanobody Magarose beads. Immunoprecipitates were analysed by immunoblotting using antibodies against GFP and V5. (C) Lysates from *ATL2* KO HeLa cells expressing 3×Flag‐ATL2 were subjected to affinity isolation using immobilized GST or GST‐KIF5B. Immunoblots and Coomassie blue‐stained gels are shown, probed with an antibody against Flag. (D) Representative images of COS‐7 cells stained with antibodies against α‐tubulin (cyan) and TOM20 (magenta): wild‐type (WT), *ATL2* KO, *KIF5B* KO, *ATL2/KIF5B* double knockout (DKO), *ATL2* KO reconstituted with ATL2, and *ATL2/KIF5B* DKO reconstituted with ATL2. Scale bar, 10 µm. (E) Mitochondrial MDR in cells as shown in (D). *n* = 104, 105, 107, 106, 107, and 105 cells from three biological replicates. Biological replicates are denoted by color, with individual MDR depicted as smaller points. Data are presented as mean ± s.d. across biological replicates. Statistical analyses were performed using ordinary one‐way ANOVA followed by Tukey's multiple comparisons test.

To test whether ATL2 depends on KIF5B to regulate mitochondrial transport, we generated *KIF5B* KO and *ATL2/KIF5B* double‐knockout (DKO) COS‐7 and HeLa cell lines using CRISPR/Cas9 approach (Figure S2F). In both cell lines, loss of either ATL2 or KIF5B caused comparable perinuclear mitochondrial clustering. The DKO cells showed no further enhancement of this phenotype (Figure 2D,E and Figure S2G,H). While ATL2 re‐expression rescued mitochondrial distribution in *ATL2* KO cells, it did not in the *ATL2/KIF5B* DKO background (Figure 2D,E and Figure S2G,H). This observation suggests that ATL2 operates through a KIF5B‐dependent pathway to control mitochondrial positioning.

### 2.3 ATL2 Cooperates With the TRAK1 Adaptor to Orchestrate Mitochondrial Distribution

TRAK1 and TRAK2 are key adaptors for mitochondrial transport, with TRAK1 preferentially binding KIF5B, which is critical for anterograde mitochondrial trafficking [5, 7]. TRAK1 loss or dominant‐negative mutant expression disrupts mitochondrial transport [9]. To explore the functional link between ATL2 and TRAK1, we examined their interaction. Co‐immunoprecipitation assays confirmed that endogenous and overexpressed ATL2 associate with TRAK1 (Figure 3A,B and Figure S3A). Further, in vitro pull‐down assays using purified GST‐TRAK1 and the His‐tagged cytosolic domain of ATL2 (6×His‐cytoATL2, residues 1–476) suggested a direct physical interaction between the two proteins (Figure 3C). Domain mapping analyses (Figure S3B–E) revealed that ATL2 interacts with TRAK1 via its GTPase domain (residues 57–373), whereas TRAK1 binds ATL2 through a C‐terminal region (residues 658–953), a site distinct from those mediating TRAK1 interactions with KIF5B or the mitochondrial receptor MIRO1 [5, 43]. Among ATL family homologs, ATL2 and ATL1 interacted with TRAK1, whereas ATL3 did not (Figure 3D and Figure S3F). This binding specificity is consistent with our observations that *ATL2* KO caused perinuclear mitochondrial clustering, whereas *ATL3* KO did not (Figure 1B and Figure S1B), indicating that TRAK1 binding correlates with ATL protein function in mitochondrial distribution. In addition, overexpressed TRAK1 colocalized with ATL2 in cells (Figure S3G), supporting their physiological association. Moreover, we used a proximity ligation assay to visualize the association between TRAK1 and the ER. The ER anchoring of TRAK1 was markedly weakened following ATL2 deletion (Figure 3E,F), indicating that ATL2 is essential for TRAK1 association with the ER.

**FIGURE 3:**
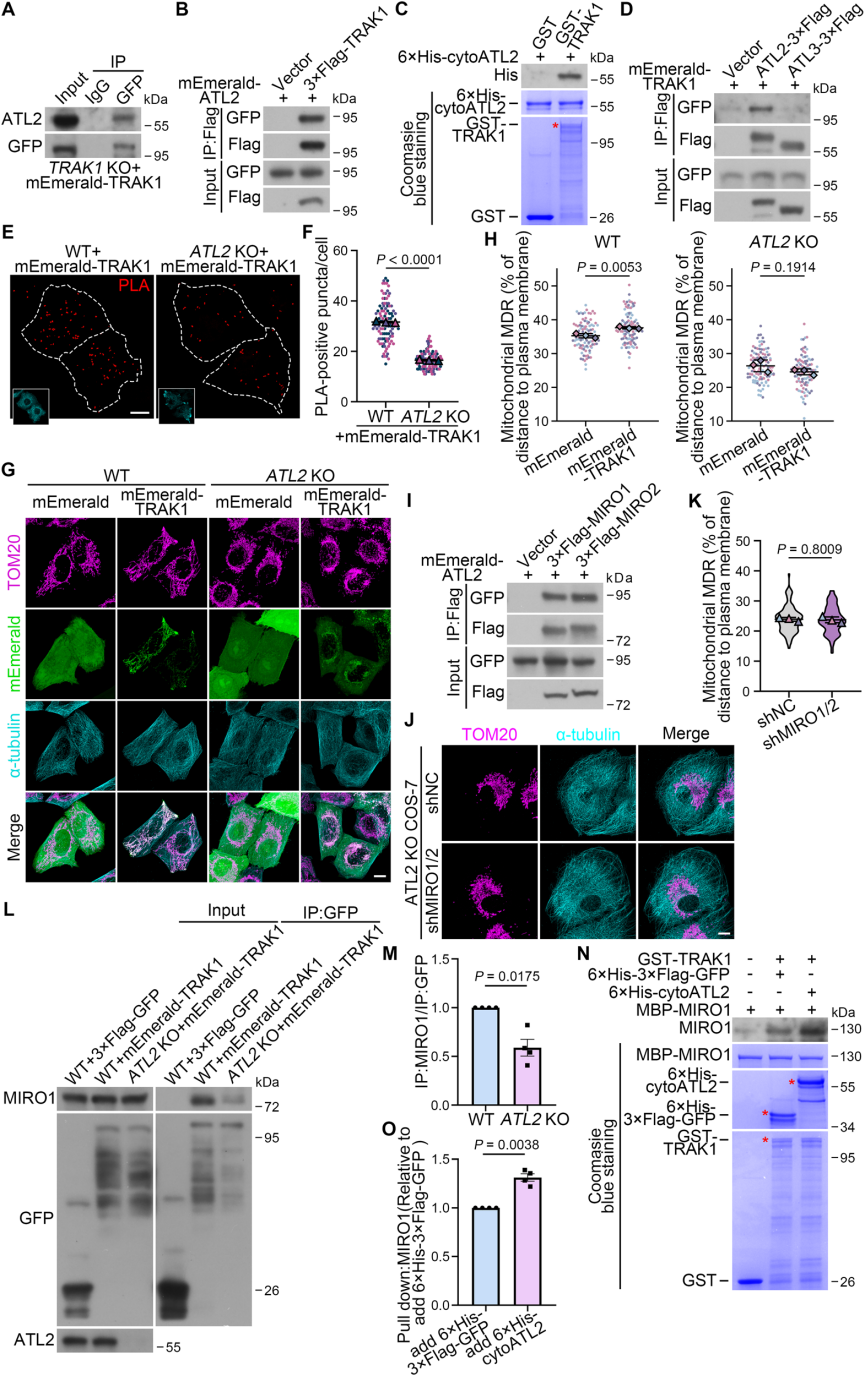
ATL2 promotes the interaction between TRAK1 and MIRO1. (A) Lysates from *TRAK1* knockout (KO) HeLa cells stably reconstituted with mEmerald‐TRAK1 were subjected to immunoprecipitation (IP) using control IgG or anti‐GFP antibody‐conjugated beads. Immunoprecipitates were analysed by immunoblotting using antibodies against GFP and ATL2. (B) HEK293T cells co‐transfected with mEmerald‐ATL2 and either 3×Flag‐TRAK1 or a control vector were subjected to immunoprecipitation using anti‐Flag M2 affinity gels. Immunoprecipitates were analysed by immunoblotting using antibodies against Flag and GFP. (C) Purified 6×His‐cytoATL2 (residues 1–476) was subjected to affinity isolation using immobilized GST or GST‐TRAK1. Immunoblots and Coomassie blue‐stained gels are shown, probed with an antibody against His. Asterisk, GST‐TRAK1. (D) HEK293T cells co‐transfected with mEmerald‐TRAK1 and either ATL2‐3×Flag or ATL3‐3×Flag were subjected to immunoprecipitation using anti‐Flag M2 affinity gels. Immunoprecipitates were analysed by immunoblotting using antibodies against Flag and GFP. (E) Proximity ligation assay (PLA) detecting the endogenous interaction between TRAK1 and calnexin in wild‐type (WT) and *ATL2* KO HeLa cells reconstituted with mEmerald‐TRAK1. Cell outlines (white dotted lines) and mEmerald‐TRAK1 (cyan) are indicated. Scale bar, 10 µm. (F) Quantification of PLA puncta per cell as shown in (E). *n* = 105 and 106 cells from three biological replicates. Biological replicates are denoted by color, with individual PLA puncta depicted as smaller points. Data are presented as mean ± s.d. across biological replicates. (G) WT or *ATL2* KO HeLa cells were transfected with mEmerald‐TRAK1 or a control vector and stained with antibodies against α‐tubulin (cyan) and TOM20 (magenta). Scale bar, 10 µm. (H) Mitochondrial MDR in cells as shown in (G). *n* = 106, 106, 107, and 100 cells from three biological replicates. Biological replicates are denoted by color, with individual MDR depicted as smaller points. Data are presented as mean ± s.d. across biological replicates. (I) HEK293T cells co‐transfected with mEmerald‐ATL2 and either 3×Flag‐MIRO1 or 3×Flag‐MIRO2 were subjected to immunoprecipitation using anti‐Flag M2 affinity gels. Immunoprecipitates were analysed by immunoblotting using antibodies against Flag and GFP. (J) Representative images of *ATL2* KO COS‐7 cells with or without additional depletion of MIRO1/2, stained with antibodies against α‐tubulin (cyan) and TOM20 (magenta). Scale bar, 10 µm. (K) Mitochondrial MDR in cells as shown in (J). *n* = 107 and 115 cells from three biological replicates. Biological replicates are denoted by color. Data are presented as mean ± s.d. across biological replicates. (L) Lysates from WT or *ATL2* KO HeLa cells stably expressing mEmerald‐TRAK1, or WT cells expressing 3×Flag‐GFP, were incubated with anti‐GFP nanobody magarose beads. Immunoprecipitates were analysed by immunoblotting using antibodies against GFP, MIRO1, and ATL2. (M) Quantification of relative intensity in (L), with data from four biological replicates presented as mean ± s.e.m. (N) In vitro pull‐down assay with purified proteins. Immobilized GST or GST‐TRAK1 was incubated with MBP‐MIRO1 and 6×His‐cytoATL2 or 6×His‐3×Flag‐GFP, followed by immunoblotting for MIRO1 and Coomassie staining (asterisks, key protein bands). (O) Quantification of relative MIRO1 intensity as shown in (N), with data from four biological replicates presented as mean ± s.e.m. Statistical analyses were performed using two‐tailed unpaired *t*‐tests with (M and O) or without (F, H, and K) Welch's correction.

To determine the functional relationship between ATL2 and TRAK1 in mitochondrial transport, we overexpressed TRAK1 in WT and *ATL2* KO HeLa cells and assessed mitochondrial distribution. In WT cells, TRAK1 overexpression significantly increased mitochondrial distribution at the cell periphery (Figure 3G,H). This effect was abolished in ATL2‐deficient cells (Figure 3G,H). These results indicate that ATL2 is required for TRAK1 to promote mitochondrial transport to the plasma membrane.

To determine whether ATL2 regulates the TRAK1–KIF5B interaction, co‐immunoprecipitation assays were performed in TRAK1‐expressing WT and *ATL2* KO HeLa cells. The association between TRAK1 and KIF5B remained unchanged upon ATL2 depletion (Figure S3H). Conversely, the interaction between ATL2 and KIF5B depended on TRAK1, as TRAK1 depletion abolished ATL2–KIF5B binding (Figure S3I), indicating that ATL2 associates with KIF5B indirectly via TRAK1.

### 2.4 ATL2 Promotes the Interaction Between TRAK1 and MIRO1

MIRO proteins, which anchor to the outer mitochondrial membrane and recruit TRAK adaptors [10, 14], co‐immunoprecipitated with overexpressed ATL2 (Figure 3I). To determine whether ATL2 and MIROs operate in a common pathway, MIRO1 and MIRO2 were depleted in *ATL2* KO cells (Figure S3J). This double deficiency did not exacerbate the mitochondrial distribution defects in *ATL2* KO cells (Figure 3J,K and Figure S3K,L), placing ATL2 and MIROs within the same functional pathway. We next asked whether ATL2 regulates the interactions between TRAK1 and MIROs. Co‐immunoprecipitation assays in TRAK1‐expressing WT and ATL2 KO HeLa cells showed a marked decrease in the TRAK1–MIRO1 interaction upon ATL2 loss, despite unchanged TRAK1–MIRO2 association (Figure 3L,M and Figure S3H). This specificity was confirmed in vitro, where ATL2 directly increased the binding between recombinant TRAK1 and MIRO1 (Figure 3N,O). Thus, ATL2 is specifically required to stabilize the TRAK1–MIRO1 interaction.

### 2.5 ATL2 is Required for Maintaining ER–Mitochondria Contact Sites

Since ATL2 is an ER membrane protein, we reasoned that it might regulate mitochondrial transport via ER–mitochondria contact sites. To test this, we performed density gradient centrifugation [44], and confirmed an accumulation of ATL2 in MAM fractions from mouse liver (Figure 4A). To provide ultrastructural confirmation, we used immunoelectron microscopy, which verified that ATL2 localizes to ER–mitochondria contact sites (Figure 4B).

**FIGURE 4:**
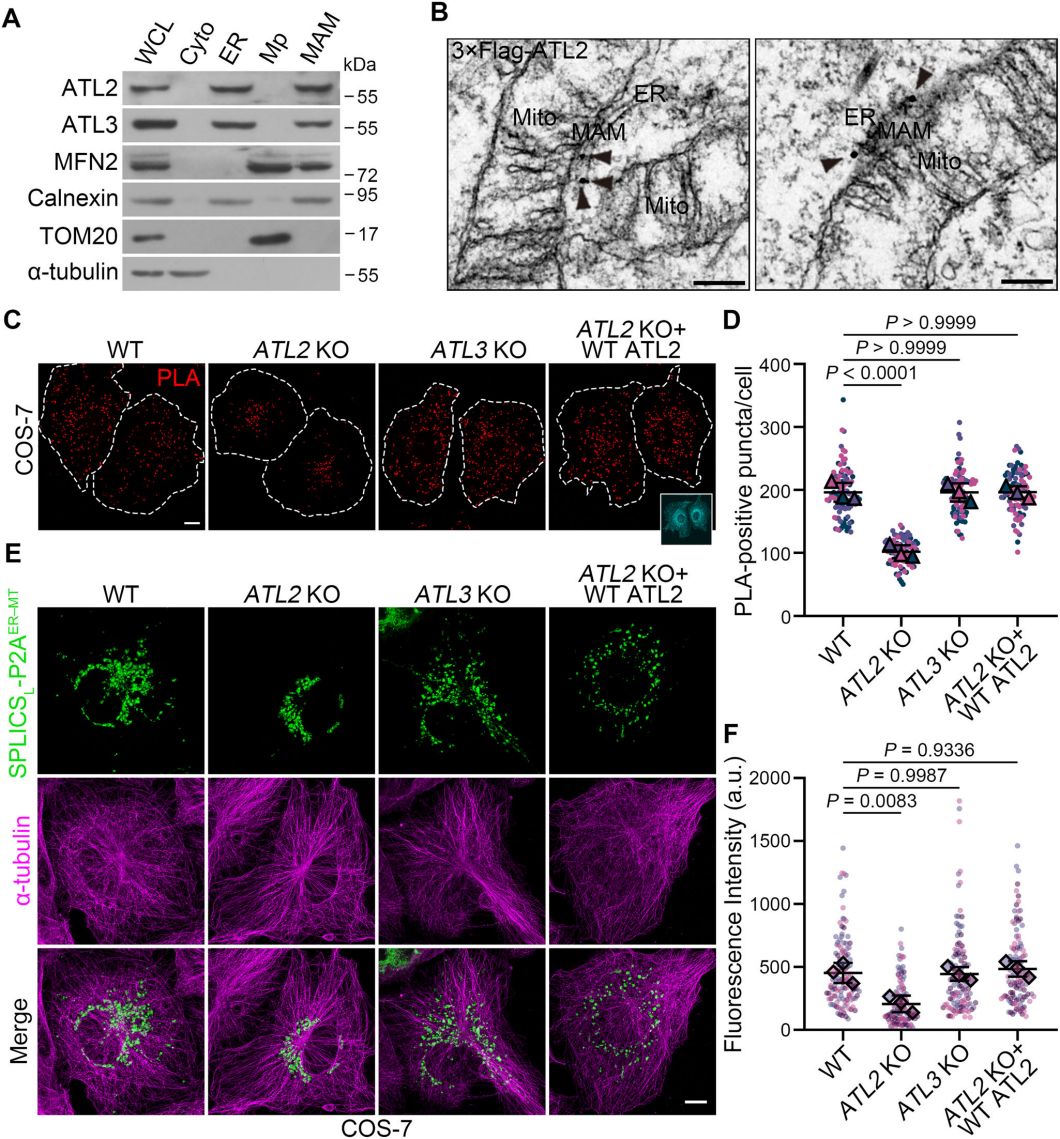
ATL2 is required for maintaining ER–mitochondria contact sites. (A) Immunoblot analysis of subcellular fractions from mouse liver. WCL, whole‐cell lysate; Cyto, cytosol; ER, endoplasmic reticulum; Mp, purified mitochondria; MAM, mitochondria‐associated ER membranes. (B) Immunogold electron microscopy of *ATL2* knockout (KO) HeLa cells stably expressing 3×Flag‐ATL2. Arrowheads mark gold particles labelling 3×Flag‐ATL2 at the MAM. Scale bar, 200 nm. (C) PLA detecting the endogenous interaction between TOM20 and calnexin in wild‐type (WT), *ATL2* KO, *ATL3* KO and *ATL2* KO reconstituted with ATL2 COS‐7 cells. Cell outlines (white dotted lines) and expressed ATL2 (cyan) are indicated. Scale bar, 10 µm. (D) Quantification of PLA puncta per cell as shown in (C). *n* = 95, 91, 93, and 91 cells from three biological replicates. Biological replicates are denoted by color, with individual PLA puncta depicted as smaller points. Data are presented as mean ± s.d. across biological replicates. (E) Representative images of WT, *ATL2* KO, *ATL3* KO, and *ATL2* KO reconstituted with ATL2 COS‐7 cells expressing SPLICS_L_‐P2A^ER‐MT^ (green) and stained with antibodies against α‐tubulin (magenta). Scale bar, 10 µm. (F) Quantification of green fluorescence intensity per cell area as shown in (E). *n* = 120, 112, 120, and 112 cells from three biological replicates. Biological replicates are denoted by color, with individual data points depicted as smaller points. Data are presented as mean ± s.d. across biological replicates. In (D) and (F), statistical analyses were performed using ordinary one‐way ANOVA followed by Tukey's multiple comparisons test.

We next performed PLA using antibodies against the ER protein calnexin and the mitochondrial outer membrane protein TOM20 to examine the effects of ATL2 on ER–mitochondria contact sites. Deletion of ATL2, but not ATL3, significantly reduced the number of PLA‐positive puncta, an effect that was rescued by ATL2 re‐expression (Figure 4C,D and Figure S4A,B). In addition, we validated the role of ATL2 in regulating ER–mitochondria contact sites using a split‐GFP‐based contact site sensor (SPLICS) system [45]. ATL2 loss, in contrast to ATL3, markedly decreased the SPLICS signal, a reduction that was reversed upon ATL2 reintroduction (Figure 4E,F and Figure S4C,D). Together, these results establish a specific requirement for ATL2 in maintaining ER–mitochondria contact sites.

To test whether physical ER‐mitochondrial proximity alone could restore mitochondrial transport, we generated two artificial mitochondria–ER tethers with distinct spacer lengths by modifying the SPLICS system [45]. Expression of either tether successfully restored MAM abundance in *ATL2* KO cells (Figure S4E,F), yet neither construct rescued the perinuclear mitochondrial clustering phenotype (Figure S4G,H). These results indicate that although the structural integrity of ER–mitochondria contact sites is essential for their function, artificially restoring physical contacts alone does not compensate for the loss of ATL2.

Calcium (Ca^2+^) transfer from the ER to mitochondria critically depends on ER–mitochondria contact sites [19, 46]. Consistent with compromised ER–mitochondrial calcium communication, live‐cell calcium imaging revealed blunted mitochondrial Ca^2+^ uptake following histamine stimulation in *ATL2* KO HeLa cells compared with WT cells (Figure S4I–K). However, inhibiting mitochondrial calcium uptake with KB‑R7943, an inhibitor of the mitochondrial calcium uniporter (MCU) [47], failed to recapitulate the perinuclear mitochondrial clustering phenotype in WT HeLa cells (Figure S4L,M). Thus, the aberrant mitochondrial distribution caused by ATL2 loss is not a consequence of impaired mitochondrial Ca^2+^ import.

### 2.6 The ATL2–MFN2 Interaction Is Required for Mitochondrial Transport

Beyond its established role in mediating ER–mitochondria tethering [21], MFN2 is also linked to the regulation of mitochondrial transport [48]. We generated *MFN1* or *MFN2* KO lines in COS‐7 and HeLa cells (Figure S5A) and validated that loss of MFN2, but not MFN1, caused perinuclear clustering of mitochondria (Figure S5B,C). We therefore investigated whether MFN2 collaborates with ATL2 to regulate mitochondrial transport. Co‐immunoprecipitation experiments in HEK293T cells confirmed the interaction between ATL2 and MFN2 (Figure 5A). This binding was specific to ATL2 and ATL1, as MFN2 did not bind ATL3 (Figure 5B and Figure S5D). The interaction was further validated under endogenous conditions and shown to be direct by in vitro pull‐down assays (Figure 5C,D and Figure S5E,F). Next, immunoprecipitation analyses revealed that ATL2 interacted with MFN2 through its GTPase domain (residues 57–373) (Figure S5G).

**FIGURE 5:**
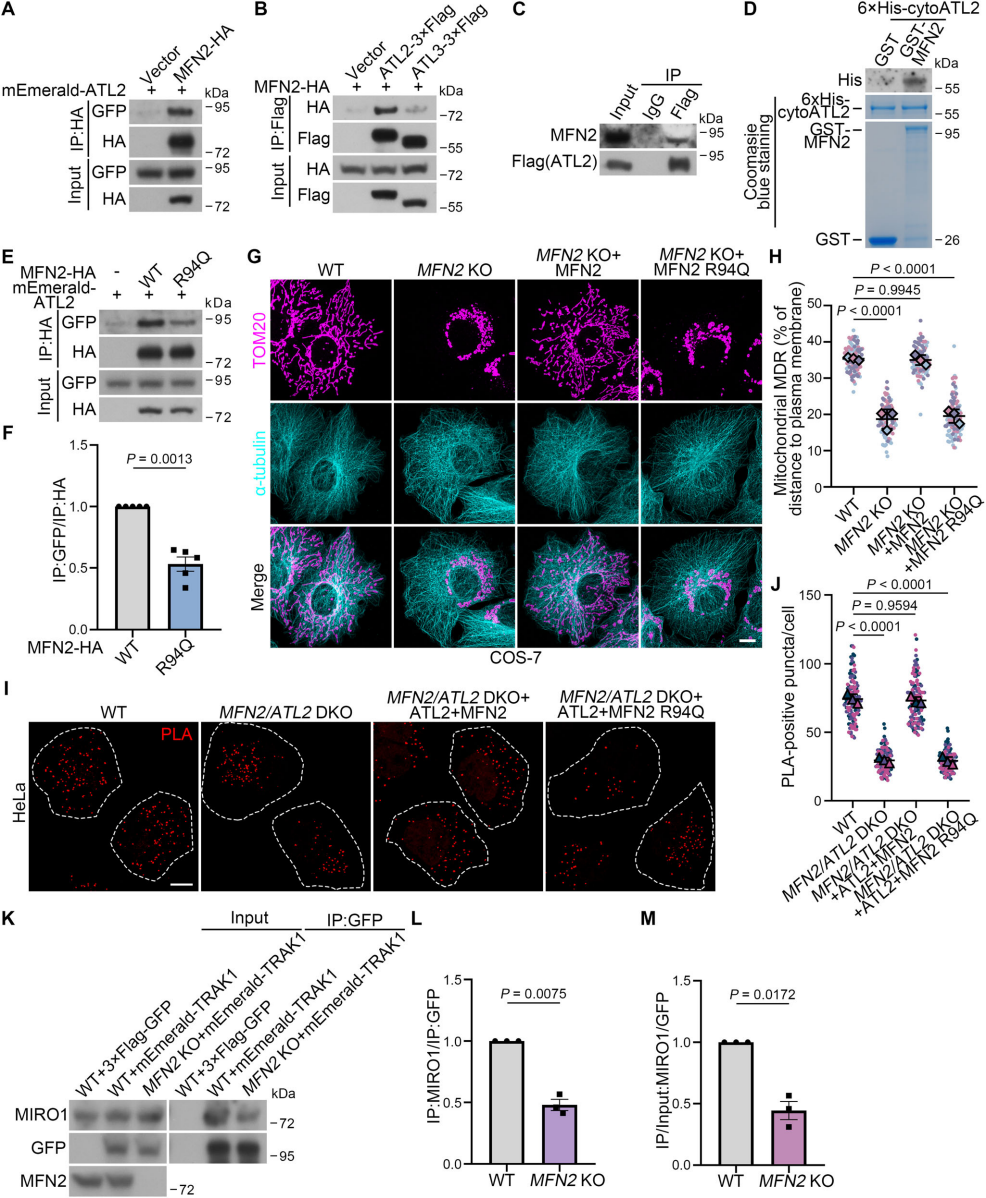
The ATL2–MFN2 interaction is required for mitochondrial transport. (A) HEK293T cells co‐transfected with mEmerald‐ATL2 and either MFN2‐HA or a control vector were subjected to immunoprecipitation (IP) using anti‐HA nanobody Magarose beads. Immunoprecipitates were analysed by immunoblotting using antibodies against HA and GFP. (B) HEK293T cells co‐transfected with MFN2‐HA with either ATL2‐3×Flag or ATL3‐3×Flag were subjected to immunoprecipitation using anti‐Flag M2 affinity gels. Immunoprecipitates were analysed by immunoblotting using antibodies against Flag and HA. (C) Lysates from *ATL2* knockout (KO) HeLa cells stably reconstituted with 3×Flag‐ATL2 were subjected to immunoprecipitation using control IgG or anti‐Flag antibody‐conjugated beads. Immunoprecipitates were analysed by immunoblotting using antibodies against Flag and MFN2. (D) Purified 6×His‐cytoATL2 (residues 1–476) was subjected to affinity isolation using immobilized GST or GST‐MFN2. Immunoblots and Coomassie blue‐stained gels are shown, probed with an antibody against His. (E) HEK293T cells co‐transfected with mEmerald‐ATL2, and either MFN2‐HA or MFN2 R94Q‐HA were subjected to immunoprecipitation using anti‐HA nanobody magarose beads. Immunoprecipitates were analysed by immunoblotting using antibodies against HA and GFP. (F) Quantification of relative intensity as shown in (E), with data from five biological replicates presented as mean ± s.e.m. (G) Representative images of wild‐type (WT), *MFN2* KO, and *MFN2* KO COS‐7 cells reconstituted with WT MFN2 or MFN2 R94Q stained with antibodies against α‐tubulin (cyan) and TOM20 (magenta). Scale bar, 10 µm. (H) Mitochondrial MDR in cells as shown in (G). *n* = 107, 106, 107, and 107 cells from three biological replicates. Biological replicates are denoted by color, with individual MDR values depicted as smaller points. Data are presented as mean ± s.d. across biological replicates. (I) PLA detecting the endogenous interaction between TOM20 and calnexin in WT, *MFN2* KO, *MFN2/ATL2* double knockout (DKO), and DKO HeLa cells reconstituted with ATL2 together with either WT MFN2 or MFN2 R94Q. Cell outlines (white dotted lines) are indicated. Scale bar, 10 µm. (J) Quantification of PLA puncta per cell as shown in (I). *n* = 125 cells from three biological replicates. Biological replicates are denoted by color, with individual PLA puncta depicted as smaller points. Data are presented as mean ± s.d. across biological replicates. (K) Lysates from WT or *MFN2* KO HeLa cells stably expressing mEmerald‐TRAK1, or WT cells expressing 3×Flag‐GFP, were incubated with anti‐GFP nanobody magarose beads. Immunoprecipitates were analysed by immunoblotting using antibodies against GFP, MIRO1, and MFN2. (L) Quantification of relative intensity as shown in (K), with data from three biological replicates presented as mean ± s.e.m. (M) Quantification of relative intensity as shown in Figure S5L, with data from three biological replicates presented as mean ± s.e.m. Statistical analyses were performed using two‐tailed unpaired *t*‐tests with Welch's correction (F, L and M) and ordinary one‐way ANOVA followed by Tukey's multiple comparisons test (H and J).

As the Charcot–Marie–Tooth type 2A‐associated MFN2 R94Q mutation disrupts ER–mitochondria contact sites and impairs mitochondrial transport [49], we investigated whether this pathogenic variant alters its interaction with ATL2. The MFN2 R94Q mutant displayed substantially reduced binding to mEmerald‐ATL2 than WT MFN2 in co‐immunoprecipitation assays (Figure 5E,F). The MFN2 R94Q mutant consistently failed to restore normal mitochondrial distribution in *MFN2* KO and *MFN2/ATL2* DKO cells, unlike WT MFN2 (Figure 5G,H and Figure S5H–K). These results indicate that the ATL2–MFN2 interaction is essential for proper mitochondrial distribution.

We hypothesized that the ATL2–MFN2 complex acts as a tether between the ER and mitochondria. To test this, we used a PLA to quantify ER–mitochondria contact sites. Deletion of both proteins in HeLa cells significantly reduced PLA signals, a phenotype rescued by WT ATL2 and WT MFN2, but not by the interaction‐deficient MFN2 R94Q mutant (Figure 5I,J). Thus, a functional ATL2–MFN2 complex is required to maintain ER–mitochondria contact sites. We next investigated whether MFN2, through its role in ER–mitochondria tethering, influences the TRAK1–MIRO1 interaction. MFN2 deletion weakened the TRAK1–MIRO1 interaction (Figure 5K,L). Furthermore, the interaction between ATL2 and MIRO1 was inhibited upon MFN2 loss (Figure 5M and Figure S5L). However, loss of MFN2 did not affect the ATL2–TRAK1 interaction (Figure S5M,N). Together, these results reveal that the ATL2–MFN2 tether couples ER–mitochondria contact sites to the mitochondrial transport machinery by stabilizing the TRAK1–MIRO1 interaction, thereby making it essential for proper mitochondrial distribution.

### 2.7 Hypoxia Induces ATL2 Degradation and Leads to Clustered Mitochondria

Perinuclear clustering of mitochondria has been observed in capillary endothelial cells of rats in hypoxia [50], yet the underlying molecular mechanism remains unclear. In COS‐7 and HeLa cells, hypoxia‐induced perinuclear clustering of mitochondria coincided with a specific decrease in ATL2 protein levels (Figure 6A–D and Figure S6A–D); whereas the expression of ATL3, calnexin, and TOM20 remained stable (Figure 6C,D and Figure S6C,D), suggesting that ATL2 degradation is a potential mechanism. Hypoxia‐induced ATL2 degradation was prevented by the proteasome inhibitor MG132 but not by the autophagy inhibitor chloroquine (Figure 6E and Figure S6E), indicating that it is ubiquitin‐proteasome‐mediated. This was further supported by increased ATL2 ubiquitination under hypoxic conditions, which was increased by MG132 (Figure 6F), demonstrating that hypoxia triggers proteasome‐dependent degradation of ATL2.

**FIGURE 6:**
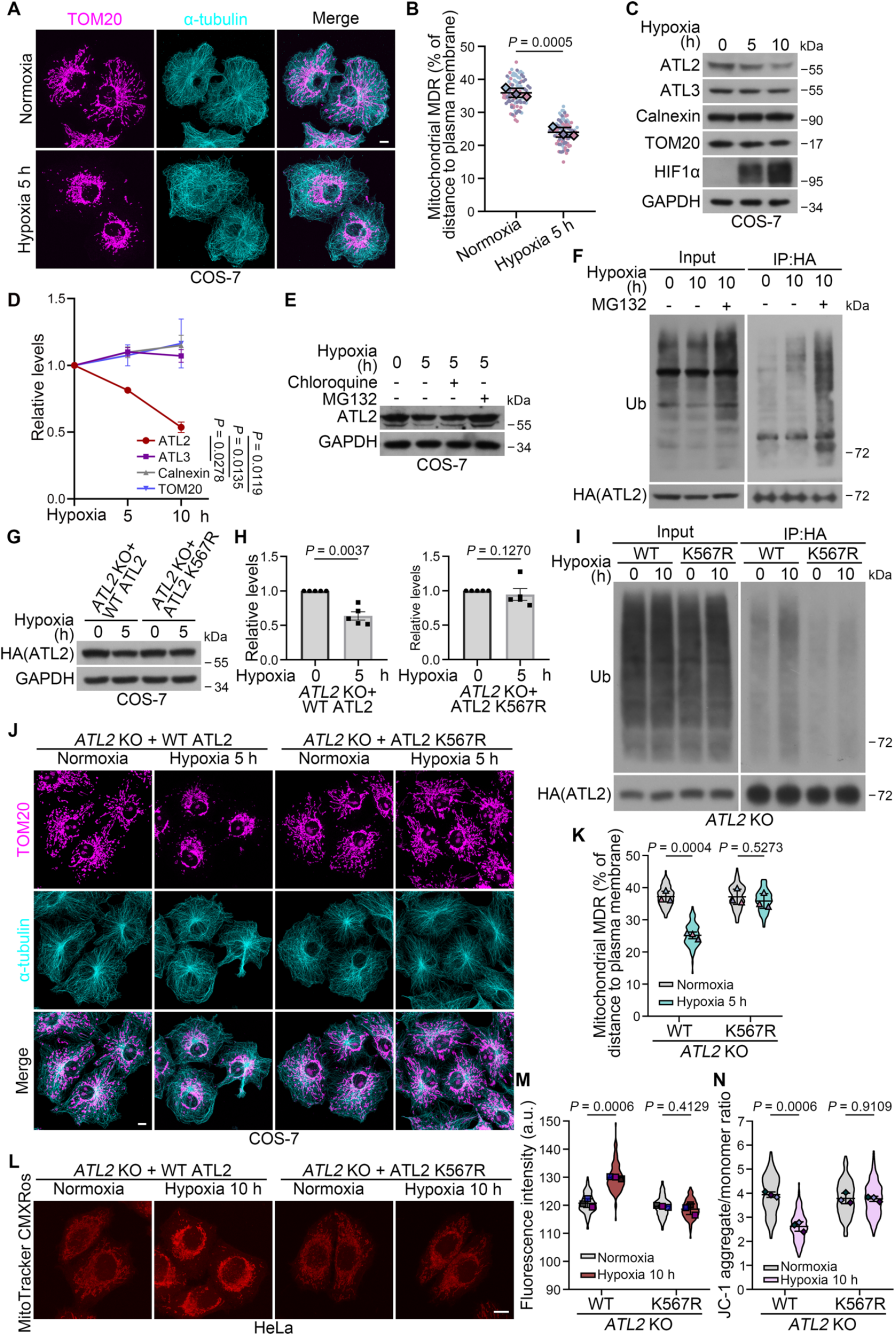
Hypoxia induces ATL2 degradation, leading to mitochondrial clustering. (A) Representative images of COS‐7 cells exposed to 1% O_2_ for the indicated times and stained with antibodies against α‐tubulin (cyan) and TOM20 (magenta). Scale bar, 10 µm. (B) Mitochondrial MDR in cells as shown in (A). *n* = 102 cells from three biological replicates. Biological replicates are denoted by color, with individual MDR values depicted as smaller points. Data are presented as mean ± s.d. across biological replicates. (C) Immunoblot analysis of ATL2, ATL3, calnexin, and TOM20 in COS‐7 cells exposed to 1% O_2_ for the indicated times. (D) Quantification of relative protein levels following 10 h of hypoxia as shown in (C), with data from three biological replicates presented as mean ± s.e.m. (E) Immunoblot analysis of ATL2 in COS‐7 cells exposed to 1% O_2_ for the indicated times in the presence of MG132 (10 µM) or chloroquine (10 µM). (F) Lysates from *ATL2* knockout (KO) HeLa cells stably expressing HA‐ATL2 exposed to 1% O_2_ for the indicated times with or without MG132 (10 µM) were subjected to immunoprecipitation (IP) using anti‐HA beads, followed by immunoblotting using antibodies against HA and ubiquitin (Ub). (G) Immunoblot analysis of HA protein levels in *ATL2* KO COS‐7 cells stably expressing HA‐WT ATL2 or HA‐ATL2 K567R, exposed to 1% O_2_ for the indicated times. (H) Quantification of relative protein levels as shown in (G), with data from five biological replicates presented as mean ± s.e.m. (I) Lysates from *ATL2* KO HeLa cells stably expressing HA‐WT ATL2 or HA‐ATL2 K567R, exposed to 1% O_2_ for 0 or 10 h, were subjected to immunoprecipitation using anti‐HA beads, followed by immunoblotting using antibodies against HA and Ub. (J) Representative images of *ATL2* KO COS‐7 cells stably expressing WT ATL2 or the ATL2 K567R mutant, exposed to 1% O_2_ for 0 or 5 h, stained with antibodies against α‐tubulin (cyan) and TOM20 (magenta). Scale bar, 10 µm. (K) Mitochondrial MDR in cells as shown in (J). *n* = 105, 105, 104, and 105 cells from three biological replicates. Biological replicates are denoted by color. Data are presented as mean ± s.d. across biological replicates. (L) Representative images of *ATL2* KO HeLa cells stably expressing WT ATL2 or the ATL2 K567R mutant, exposed to 1% O_2_ for 0 or 10 h, stained with MitoTracker Red CMXRos (100 µM). Scale bar, 10 µm. (M) Quantification of fluorescence intensity per cell area as shown in (L). *n* = 116, 116, 115, and 118 cells from three biological replicates. Biological replicates are denoted by color. Data are presented as mean ± s.d. across biological replicates. (N) Quantification of the JC‐1 aggregate/monomer ratio as shown in Figure S7E. *n* = 150 cells from three biological replicates. Biological replicates are denoted by color. Data are presented as mean ± s.d. across biological replicates. Statistical analyses were performed using ordinary one‐way ANOVA followed by Tukey's multiple comparisons test (D) and two‐tailed unpaired *t*‐tests with (H) or without (B, K, M, and N) Welch's correction.

To identify the residue responsible for hypoxia‐induced degradation, we performed mass spectrometry‐based ubiquitination profiling and identified lysine 567 (K567) as a potential hypoxia‐induced ubiquitination site in ATL2 (Figure S6F). Mutation of K567 to arginine (K567R) inhibited ATL2 degradation in response to hypoxic treatment (Figure 6G,H and Figure S7A,B). Consistent with this, ubiquitination of the K567R mutant was decreased in hypoxia compared with WT ATL2 (Figure 6I). The K567R mutation also prevented the hypoxia‐induced perinuclear clustering of mitochondria (Figure 6J,K and Figure S7C,D). These data demonstrate that ubiquitination of K567 is the key signal triggering ATL2 degradation and subsequent mitochondrial perinuclear clustering in hypoxia.

We next evaluated mitochondrial function in hypoxia. Hypoxia induced a marked increase in mitochondrial reactive oxygen species (ROS) in cells expressing WT ATL2, whereas the K567R mutant effectively suppressed this ROS elevation (Figure 6L,M). Concomitantly, the hypoxia‐induced reduction in mitochondrial membrane potential was rescued by the K567R mutant (Figure 6N and Figure S7E).

### 2.8 SYVN1 is Responsible for ATL2 Ubiquitination in Hypoxia

ER‐localized E3 ubiquitin ligase synoviolin 1 (SYVN1) ubiquitinates ATL family proteins [51]. Co‐immunoprecipitation assays confirmed the association between SYVN1 and ATL2 (Figure 7A,B). In vitro pull‐down assays showed that purified GST‐SYVN1 directly bound to ATL2, demonstrating a direct interaction between SYVN1 and ATL2 (Figure 7C). Overexpression of WT SYVN1, but not its catalytically inactive C329S mutant [51], markedly increased ATL2 ubiquitination (Figure 7D). The K567R mutation of ATL2 abolished SYVN1‐induced ATL2 ubiquitination (Figure 7E). Pharmacological inhibition of SYVN1 by LS‐102 [52] prevented hypoxia‐induced ubiquitination and degradation of ATL2 (Figure 7F–H). Consequently, SYVN1 inhibition rescued the aberrant perinuclear mitochondrial clustering observed under hypoxia (Figure 7I,J), demonstrating that SYVN1 is critically involved in hypoxia‐induced ATL2 degradation. Moreover, inhibition of SYVN1 effectively rescued the hypoxia‑induced reduction in ER–mitochondria contacts (Figure 7K,L). Collectively, these data establish that hypoxia promotes SYVN1‐mediated ubiquitination of ATL2 at K567, leading to its proteasomal degradation and disruption of mitochondrial distribution.

**FIGURE 7:**
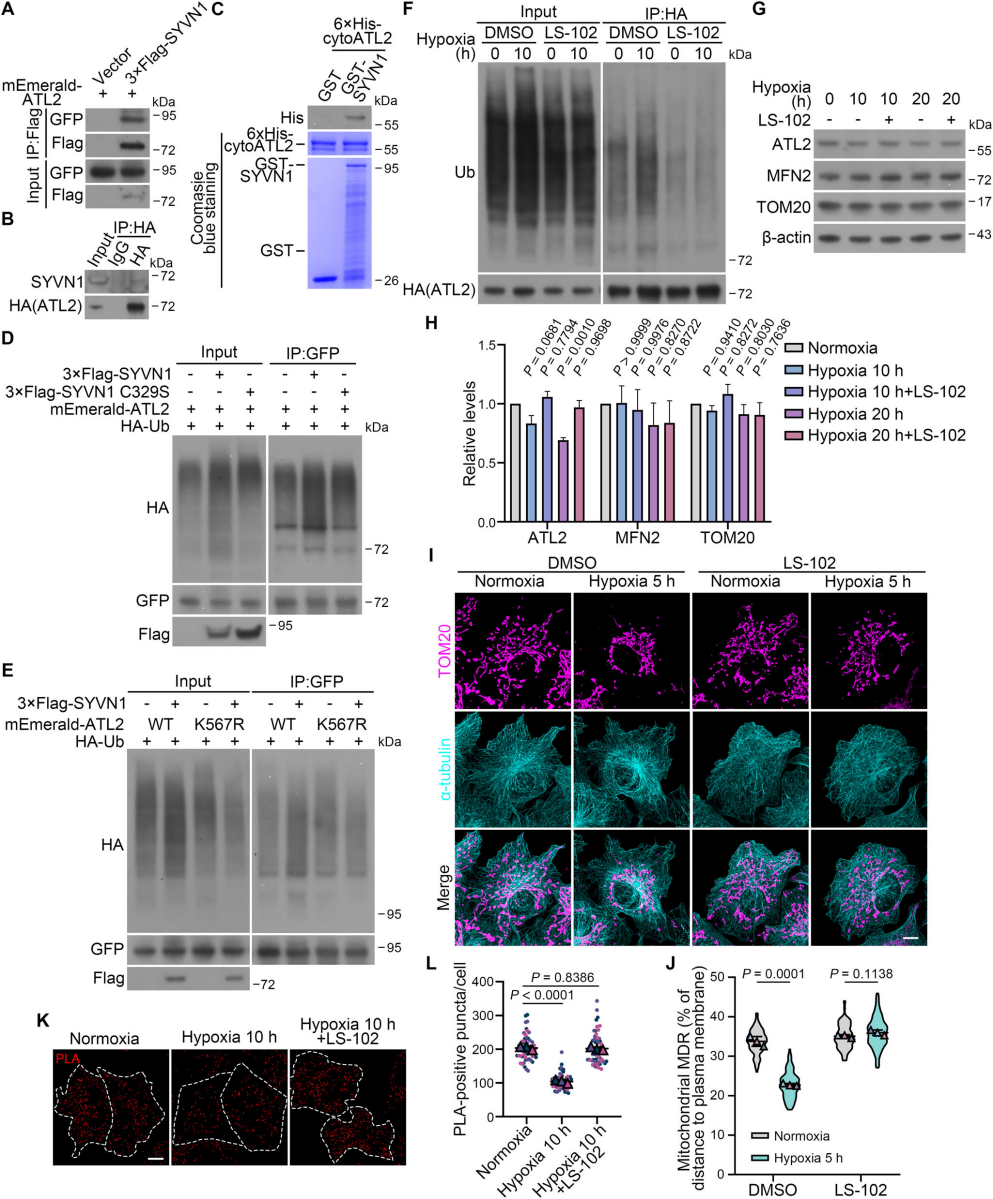
SYVN1 is responsible for ATL2 ubiquitination in hypoxia. (A) HEK293T cells co‐transfected with mEmerald‐ATL2 and either 3×Flag‐SYVN1 or a control vector were subjected to immunoprecipitation (IP) using anti‐Flag M2 affinity gels. Immunoprecipitates were analysed by immunoblotting using antibodies against Flag and GFP. (B) Lysates from *ATL2* knockout (KO) HeLa cells stably reconstituted with HA‐ATL2 were subjected to immunoprecipitation using control IgG or anti‐HA antibody‐conjugated beads. Immunoprecipitates were analysed by immunoblotting using antibodies against HA and SYVN1. (C) Purified 6×His‐cytoATL2 (residues 1–476) was subjected to affinity isolation using immobilized GST or GST‐SYVN1. Immunoblots and Coomassie blue‐stained gels are shown, probed with an antibody against His. (D) HEK293T cells transfected with mEmerald‐ATL2, HA‐Ubiquitin (Ub), together with either 3×Flag‐SYVN1 or the 3×Flag‐SYVN1 C329S mutant, were subjected to immunoprecipitation using anti‐GFP nanobody magarose beads. Immunoprecipitates were analysed by immunoblotting using antibodies against GFP, HA, and Flag. (E) HEK293T cells transfected with HA‐Ub, mEmerald‐ATL2 (WT and K567R), together with either 3×Flag‐SYVN1 or a control vector, were subjected to immunoprecipitation using anti‐GFP nanobody magarose beads. Immunoprecipitates were analysed by immunoblotting using antibodies against GFP, HA, and Flag. (F) Lysates from *ATL2* KO HeLa cells stably expressing HA‐ATL2, exposed to 1% O_2_ for the indicated times with or without LS‐102 (20 µM), were subjected to immunoprecipitation using anti‐HA beads, followed by immunoblotting using antibodies against HA and Ub. (G) Immunoblot analysis of ATL2, MFN2 and TOM20 protein levels in HeLa cells exposed to 1% O_2_ for the indicated times with or without LS‐102 (20 µM). (H) Quantification of relative protein levels as shown in (G), with data from four biological replicates presented as mean ± s.e.m. (I) Representative images of COS‐7 cells exposed to 1% O_2_ for the indicated times with or without LS‐102 (20 µM), stained with antibodies against α‐tubulin (cyan) and TOM20 (magenta). Scale bar, 10 µm. (J) Mitochondrial MDR in cells as shown in (I). *n* = 101, 101, 100, and 103 cells from three biological replicates. Biological replicates are denoted by color. Data are presented as mean ± s.d. across biological replicates. (K) PLA detecting the endogenous interaction between TOM20 and calnexin in WT HeLa cells exposed to 1% O_2_ for the indicated times with or without LS‐102 (20 µM). Cell outlines are indicated. Scale bar, 10 µm. (L) Quantification of PLA puncta per cell as shown in (K). *n* = 58 cells from three biological replicates. Biological replicates are denoted by color, with individual PLA puncta depicted as smaller points. Data are presented as mean ± s.d. across biological replicates. Statistical analyses were performed using ordinary one‐way ANOVA followed by Dunnett's multiple comparisons test (H and L) and two‐tailed unpaired *t*‐tests (J).

## 3 Discussion

ER–mitochondria contact sites are well‐established signaling hubs that mediate lipid exchange, calcium flux, and mitochondrial fission [18, 19]. Accumulating evidence hints that these junctions may also govern microtubule‐based mitochondrial trafficking. For example, these two organelles associate during movement and co‐localize on acetylated microtubules [53]. The transport regulator MIRO1 is detected at these contact sites, and its yeast ortholog Gem1 is a component of the ER–mitochondria encounter structure tethering complex [54]. Furthermore, the ER protein REEP5 interacts with MFN1/2, promoting coupled organellar movement [55]. Nevertheless, the molecular machinery underlying ER‐mediated regulation of mitochondrial transport along microtubules remained undefined. Here, we identify ATL2 as a core MAM scaffold that coordinates mitochondrial transport complex assembly. ATL2 recruits the cytosolic adaptor TRAK1 to the ER through its GTPase domain, while its interaction with mitochondrial MFN2 establishes a membrane‐tethering platform. This collaborative assembly promotes the formation of the TRAK1–MIRO1 complex to drive anterograde mitochondrial transport (Figure 8). Furthermore, we link this mechanism to the hypoxic response, in which ubiquitination at K567 dismantles ATL2, impairing mitochondrial transport and causing perinuclear clustering.

**FIGURE 8:**
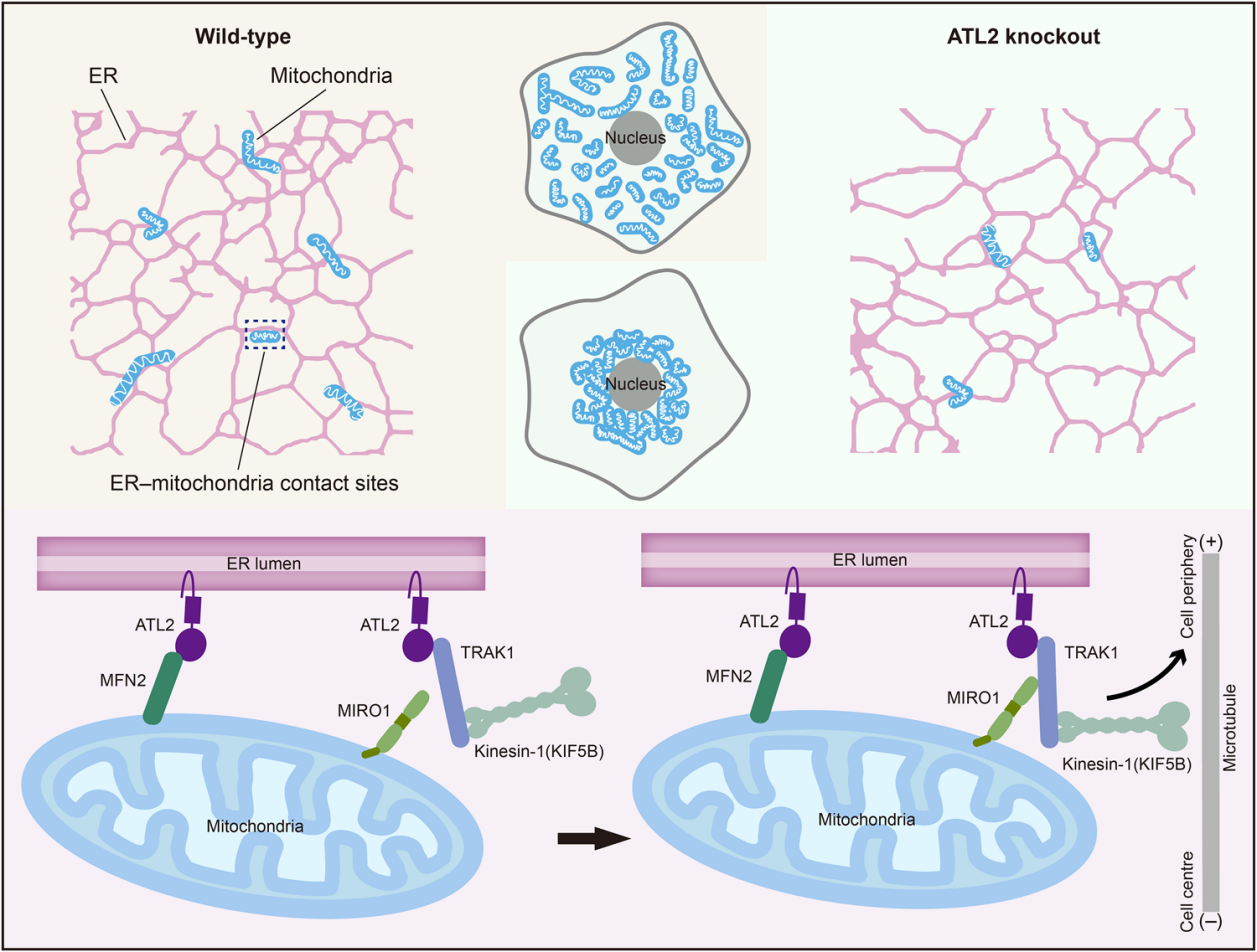
ER–mitochondria contacts orchestrate mitochondrial transport via ATL2. The ER protein ATL2 promotes the TRAK1–MIRO1 interaction by recruiting TRAK1 to the ER and by interacting with mitochondrial MFN2 at contact sites. This facilitates assembly of the transport adaptor complex, which in turn recruits the motor protein KIF5B to drive anterograde mitochondrial transport.

The ATL GTPases exhibit distinct tissue distributions. ATL1 is found predominantly in the brain, whereas ATL2 and ATL3 are more enriched in non‐neuronal tissues [26]. ATL2 expression is particularly high in muscle [32]. This expression pattern aligns with our findings that ATL2 governs mitochondrial transport and distribution, processes essential for the function of this high‐energy‐demand tissue. Despite high sequence conservation, ATL3 cannot bind TRAK1 or MFN2, whereas ATL2 interacts with both (Figures 3D and 5B); ATL1, which shares greater sequence homology with ATL2 [56], also interacts with TRAK1 and MFN2 (Figures S3F and S5D), indicating functional divergence among homologs. Mutations in the *ATL1* gene are a well‐established genetic cause of hereditary spastic paraplegia, a neurodegenerative disease [29]. In a disease‐relevant model, forebrain neurons differentiated from patient‐derived pluripotent stem cells carrying the ATL1 P342S mutation exhibit impaired mitochondrial transport [33]. Whether ATL1 regulates neuronal mitochondrial transport via an ATL2‐like mechanism remains an important question; the answers to this question will deepen our mechanistic understanding of the neurodegenerative pathology of hereditary spastic paraplegia.

Our findings position ATL2 as a molecular scaffold that coordinates assembly of the mitochondrial transport machinery. We identified a specific C‐terminal domain (residues 658–953) in TRAK1 that mediates its recruitment to the ER by ATL2 (Figure S3D,E), distinct from its MIRO1‐binding regions [43]. This spatially constrained recruitment facilitates efficient transfer of TRAK1 to mitochondrial MIRO1 at ER–mitochondria contact sites, thereby promoting transport complex assembly and driving anterograde mitochondrial transport. The essential role of this ATL2‐mediated platform is underscored by the disruption of TRAK1–MIRO1 interaction upon ATL2 depletion and its potent increase upon reconstitution with recombinant ATL2 in vitro (Figure 3L–O). We propose that the ATL2–MFN2 tether structurally aligns the organelles, enabling this precise molecular handover. It remains unclear whether TRAK1 and MFN2 compete for overlapping binding surfaces within the ATL2 GTPase domain or bind separate epitopes; future work may also uncover the upstream signals that dictate the spatiotemporal timing of TRAK1 handover to mitochondria.

The role of MFN2 as an ER–mitochondria tether, bridging organelles via homotypic or heterotypic interactions [21, 57], is conceptually appealing but limited by unresolved questions regarding its ER localization. We identified the ER‐resident GTPase ATL2 as an essential partner that functionally and physically couples with mitochondrial MFN2. First, ATL2 is enriched at ER–mitochondria contact sites, and its deletion reduces contact site abundance (Figure 4 and Figure S4). Furthermore, ATL2 directly interacts with mitochondrial MFN2 (Figure 5D). Importantly, the MFN2 R94Q pathogenic variant disrupts this interaction, providing a molecular explanation for the observed contact site deficiency (Figure 5E–J and Figure S5J,K). Together, these results identify the ATL2–MFN2 complex as a core organelle tether. Future work will elucidate the regulatory mechanisms governing this complex and its potential interactions with other tethering proteins.

## 4 Experimental Section

### 4.1 Plasmids Construction and Reagents

The plasmids related to lentivirus production were gifts from Dr. Jiang Zhengfan (College of Life Sciences, Peking University, Beijing, China). ER marker plasmid was a gift from Dr. Dong Li (Institute of Biophysics, Chinese Academy of Sciences, Beijing, China). The sequences encoding human ATL2, ATL3, TRAK1, MFN2, MIRO1, KIF5B, and SYVN1 were amplified from a HEK293T cell cDNA library by PCR and cloned into the pSIN, p3×FLAG‐CMV‐7.1 (Sigma‐Aldrich, E7533), p3×FLAG‐CMV‐14 (Sigma‐Aldrich, E7908), pcDNA3.1 (+) (Invitrogen, V79020), mEmerald‐C1 (Addgene, 53975), N1‐mScarlet (Addgene, 85054), pCold‐MBP (NovoPro Bioscience Inc., V012984), pET‐28a (+) (Novagen, 69864), or pGEX‐6P‐1 (GE Healthcare, 28‐9546‐48) vectors. Mutations in ATL2, MFN2, and SYVN1 were generated using PCR site‐directed mutagenesis. SPLICS Mt‐ER Long P2A (Addgene, 164107) was purchased from Addgene.

Chloroquine (C6628), MG132 (M8699), puromycin (P8833), histamine (H7125), and the Duolink In Situ PLA kit (DUO92002, DUO92004, DUO92008, DUO92013) were obtained from Sigma‐Aldrich. Blasticidin (S7419) and KB‐R7943(S4643) were obtained from Selleckchem. LS‐102 (HY‐135844) was purchased from MedChemExpress.

### 4.2 Antibodies

Primary antibodies used were rabbit polyclonal anti‐ATL2 (Proteintech, 16688‐1‐AP; immunoblot, 1:1,000), rabbit polyclonal anti‐ATL3 (Proteintech, 16921‐1‐AP; immunoblot, 1:1000), mouse monoclonal anti‐CLIMP63 (Enzo Life Sciences, ENZ‐ABS669; immunoblot, 1:1000), rabbit polyclonal anti‐Calnexin (Proteintech, 10427‐2‐AP; immunoblot, 1:5000, immunofluorescence, 1:500), mouse monoclonal anti‐Calnexin (Proteintech, 66903‐1‐Ig; immunofluorescence, 1:500), rabbit polyclonal anti‐Flag (Proteintech, 20543‐1‐AP; immunofluorescence, 1:200), mouse monoclonal anti‐Flag (Sigma‐Aldrich, F1804, clone M2; immunoblot, 1:5000, immunofluorescence, 1:500), rabbit polyclonal anti‐GFP (Proteintech, 50430‐2‐AP; immunoblot, 1:3000, immunofluorescence, 1:300), mouse monoclonal anti‐GAPDH (Proteintech, 60004‐1‐AP; immunoblot, 1:20 000), mouse monoclonal anti‐HA (Sigma‐Aldrich, H9658, clone HA‐7; immunoblot, 1:10 000), rabbit polyclonal anti‐HIF1α (Proteintech, 20960‐1‐AP; immunoblot, 1:1 000), mouse monoclonal anti‐His (Abmart, M20001; immunoblot, 1:10 000), rabbit polyclonal anti‐HSP60 (Proteintech, 15282‐1‐AP; immunoblot, 1:2000), rabbit polyclonal anti‐KIF5B (Proteintech, 21632‐1‐AP; immunoblot, 1:2000), rabbit polyclonal anti‐Lunapark (Abcam, Ab121416; immunoblot, 1:500), rabbit polyclonal anti‐MFN1 (Proteintech, 13798‐1‐AP; immunoblot, 1:1000), rabbit monoclonal anti‐MFN2 (Cell Signaling Technology, 9482S; immunoblot, 1:10 000), mouse monoclonal anti‐MIRO1 (Sigma‐Aldrich, WH0055288M1; immunoblot, 1:1000), rabbit polyclonal anti‐MIRO2 (Proteintech, 11237‐1‐AP; immunoblot, 1:1000), rabbit polyclonal anti‐RTN4 (Novus Biologicals, NB100‐56681; immunoblot, 1:1000), rabbit polyclonal anti‐SYVN1 (Proteintech, 13473‐1‐AP; immunoblot, 1:1000), mouse monoclonal anti‐TIM50 (Santa Cruz Biotechnology, sc‐393678; immunoblot, 1:1000), mouse monoclonal anti‐TOM20 (BD Biosciences, 612278; immunofluorescence, 1:500), rabbit polyclonal anti‐TOM20 (Proteintech, 11802‐1‐AP; immunoblot, 1:5000, immunofluorescence, 1:500), rabbit polyclonal anti‐ubiquitin (Proteintech, 10201‐2‐AP; immunoblot, 1:1000), mouse monoclonal anti‐V5 (Thermo Fisher Scientific, R960‐25; immunoblot, 1:5000), mouse monoclonal anti‐α‐tubulin (Sigma‐Aldrich, T6199, clone DM1A, immunoblot, 1:10 000, immunofluorescence, 1:1000), and mouse monoclonal anti‐β‐actin (Proteintech, 66009‐1‐Ig, clone 2D4H5, immunoblot, 1:20 000). Horseradish peroxidase‐conjugated goat anti‐rabbit or anti‐mouse secondary antibodies were purchased from Jackson ImmunoResearch. Alexa Fluor 488/568/647‐conjugated goat anti‐mouse IgG (H+L) or 568‐conjugated goat anti‐rabbit IgG (H+L) highly cross‐adsorbed secondary antibodies were obtained from Invitrogen.

### 4.3 Cell Culture and Transfections

COS‐7, HeLa, and HEK293T cells were grown in high‐glucose Dulbecco's modified Eagle medium (DMEM, Cellmax, CGM101.06) supplemented with 10% fetal bovine serum (FBS, Cellmax, SA201.02) under 5% CO_2_ at 37°C. For hypoxia treatment, cells were cultured in a hypoxic chamber (Billups‐Rothenberg) with 1% O_2_, 5% CO_2_, and 94% N_2_ at 37°C. Polyethylenimine (Polysciences, 23966) was used to transfect HEK293T cells. COS‐7 and HeLa cells were transfected with Lipofectamine 3000 (Thermo Fisher Scientific, L3000015) according to the manufacturer's instructions.

### 4.4 Immunoblotting

Samples were boiled at 100°C for 8 min, separated on 4%–12% Bis‐Tris gels (GenScript), and transferred to polyvinylidene difluoride membranes (Millipore, IPVH00010). Membranes were blocked with 4% (w/v) skim milk powder in Tris‐buffered saline containing 0.1% Tween‐20 (TBST) for 20 min at room temperature. After blocking, the membranes were incubated with primary antibodies diluted in blocking buffer for 1.5 h at room temperature, followed by TBST washes. Membranes were then probed with horseradish peroxidase‐conjugated secondary antibodies for 1.5 h at room temperature. Following washing with TBST, immunoreactive bands were detected by enhanced chemiluminescence and captured on x‐ray film in a darkroom. Immunoblot band intensities were quantified using ImageJ software (National Institutes of Health).

### 4.5 Immunofluorescence and Live‐Cell Imaging

For immunofluorescence, cells cultured on glass coverslips were washed twice with PBS and fixed with 4% (w/v) paraformaldehyde at 37°C for 15 min. After three washes with PBS, cells were permeabilized with 0.15% Triton X‐100 in PBS for 9 min at room temperature and then washed three times with PBS. Blocking was performed using 4% BSA in PBS for 30 min at room temperature. Cells were then incubated with primary antibodies diluted in blocking buffer for 1.5 h at room temperature. Following three PBS washes, cells were incubated with secondary antibodies for 1.5 h at room temperature, with or without DAPI (Invitrogen, D1306). After three final PBS washes, coverslips were mounted using Fluoromount‐G (SouthernBiotech, 0100–01) and stored overnight at 4°C. Images were acquired using a Leica TCS SP8 confocal system with a 63×/1.4 NA or 100×/1.4 NA oil‐immersion objective, a ZEISS LSM 980 confocal microscope with Airyscan and a 63×/1.4 NA oil‐immersion objective, or a Live SR CSU W1 spinning disk confocal system equipped with a 100×/1.4 NA oil‐immersion objective. Image analysis was performed using ZEN (Zeiss) or ImageJ.

For live‐cell imaging, COS‐7 cells in glass‐bottom dishes (Cellvis, D35‐20‐1.5‐N) were incubated with 125 nM PK Mito Deep Red (Genvivo, PKMDR‐2) at 37°C for 45 min for mitochondrial labeling. Live‐cell imaging was performed on a High Sensitivity Structured Illumination Microscope (HIS‐SIM; Guangzhou Computational Super‐resolution Biotech) equipped with a 100×/1.5 NA oil‐immersion objective, using IMAGER software in 2D‐SIM‐2 mode. Time‐lapse acquisition was performed for 10 min with 2‐s intervals. Acquired images were first reconstructed using Wiener Deconvolution to generate super‐resolution images, followed by Sparse Deconvolution with MicroscopeX FINER software.

### 4.6 Ca^2+^ Imaging

Ca^2+^ imaging was performed on WT and *ATL2* KO HeLa cells stably expressing mitochondria‑targeted R‑GECO (mito‑R‑GECO), a genetically encoded red fluorescent Ca^2+^ sensor [58]. Cells were imaged on a SpinSR spinning‑disk confocal microscope (Olympus) equipped with a 40×/0.95 NA objective. Time‑lapse images were acquired every 1 s for 280 s. Histamine was added to a final concentration of 100 µM at 30 s of imaging. Image analysis was carried out using ImageJ.

### 4.7 Quantification of Mitochondrial Distribution

Mitochondrial distribution was quantified following an established method for ER distribution [35]. Confocal images were processed in ImageJ, in which the cell center was identified from the DAPI signal (channel 1), and the cell boundary was defined using microtubule or ER markers (channel 3). Mitochondrial signals (channel 2) and reference signals were extracted and exported to MATLAB for computational analysis. The analysis generated 3600 radial segments at 0.1° intervals from the cell center to the farthest peripheral point. Fluorescence intensities of both nuclear and mitochondrial signals along each radius were normalized. The mean distribution radius (MDR) was calculated as the average distance of mitochondria between the nuclear envelope and the plasma membrane, with higher MDR values indicating increased peripheral mitochondrial localization.

### 4.8 Reactive Oxygen Species (ROS)

HeLa cells grown on coverslips were washed with PBS and stained with 100 µM MitoTracker Red CMXRos (Invitrogen, M7512) at 37°C for 30 min. After three PBS washes, cells were fixed and mounted for microscopic imaging. Representative images were acquired using a Leica TCS SP8 confocal microscope with a 100×/1.4 NA oil‐immersion objective. For quantitative analysis, images were acquired using a Live SR CSU‐W1 spinning disk confocal system equipped with a 100×/1.4 NA oil‐immersion objective. Mean fluorescence intensity of MitoTracker Red signals was quantified using ImageJ.

### 4.9 Mitochondrial Membrane Potential Assay

Cells were seeded in CellCarrier‐96 Ultra microplates (PerkinElmer, 6055302) and cultured under normoxic or hypoxic conditions. After washing with PBS, cells were incubated with 2.5 µg/mL JC‐1 (Yeasen, 40705ES03) for 20 min at 37°C. Following two PBS washes, the probe solution was replaced with fresh culture medium. Representative images were acquired using a Leica TCS SP8 confocal microscope with a 100×/1.4 NA oil‐immersion objective. For quantification, images were obtained using an Operetta CLS high‐content analysis system (PerkinElmer) equipped with a 20×/1.0 NA automated water‐immersion objective. Mean fluorescence intensity of JC‐1 aggregates (red) and monomers (green) was quantified using ImageJ.

### 4.10 Lentivirus Production and Stable Cell Lines

For lentivirus production, HEK293T cells were transfected via polyethylenimine with the packaging plasmid psPAX2, the envelope plasmid pMD2.G, and the indicated transfer plasmids. After 6 h, the medium was replaced with DMEM supplemented with 20% fetal bovine serum. Viral supernatant was harvested 48 h later, filtered through a 0.22 µm membrane (Millipore), and concentrated by adding one‐third volume of 40% (w/v) PEG8000, followed by gentle mixing overnight at 4°C. Viral particles were pelleted by centrifugation and then resuspended in a small volume of DMEM for storage at −80°C. For infection, target cells were incubated with concentrated virus and 8 µg/mL polybrene (Sigma‐Aldrich, 107689). The medium was refreshed 24 h postinfection. To generate stable cell lines, cells were selected with appropriate antibiotics starting 48 h after infection. For fluorescence‐based sorting, cells were prepared and sorted by flow cytometry (MoFlo Astrios EQ, Beckman Coulter).

### 4.11 CRISPR/Cas9 Gene Editing

Knockout cell lines in COS‐7 and HeLa cells were generated using the CRISPR/Cas9 system. The targets sequences used were: 5′‐GCTTAGATACATGTATAACA‐3′ and 5′‐AGAGTATGGAAGACTTGCGA‐3′ for ATL2; 5′‐GTGGCAGCAGCTGCCTCAAG‐3′ and 5′‐AGATCTTGATGTGGTGGTGG‐3′ for ATL3; 5′‐GCATATGGACAAACATCCTC‐3′ and 5′‐GTTATGGATACCATAGATGA‐3′ for KIF5B; 5′‐AGTGACAAAGTGCTTAAGTG‐3′ and 5′‐GGTTACCTATCCAAAGTGAG‐3′ for MFN2; 5′‐CCTAGTGGGATTGGCCATATAA‐3′ and 5′‐GCTACTGTGAAAAACATAATGG‐3′ for MFN1; 5′‐CTAAGTTATTTACCTTGTCC‐3′ for Lunapark; 5′‐GCCGCGCCCGCCATGCCCTCGG‐3′ for CLIMP63; 5′‐CATCAGCTTTAGGATATAC‐3′ for RTN4; 5′‐GTGTGATCTCCTCTTGCTGG‐3′ for TRAK1. The oligonucleotides were synthesized, cloned into the lentiCRISPRv2 vector (Addgene, 52961), and delivered via lentiviral transduction. At 48 h post‐infection, cells were selected with the appropriate antibiotic. Single‐cell clones were isolated by flow cytometry (MoFlo XDP, Beckman Coulter), expanded for approximately 2 weeks, and then transferred to 24‐well plates. A portion of each clone was used for immunoblotting analysis, while the remainder was maintained in culture. Clones exhibiting loss of the target protein, as determined by immunoblotting, were further expanded in 12‐well plates and verified by genomic sequencing.

### 4.12 Generation of Knockdown Cell Lines

Gene knockdown was achieved by lentiviral transduction of shRNA constructs cloned into the pLKO.1‐puro vector (Addgene, 8453). The targeting sequences used to knock down the genes were 5′‐GCAATCCCAAATCCTTTGAAT‐3′ for MIRO1 and 5′‐CCCAGAATTCTCAGGGCTCTA‐3′ for MIRO2. Following infection, cells were selected with 2 µg/mL puromycin, and knockdown efficiency was validated by immunoblotting.

### 4.13 Co‐Immunoprecipitation

HeLa or HEK293T cells were lysed in ice‐cold buffer (25 mM HEPES, pH 7.4, 0.5% Triton X‐100, 150 mM KOAc, and 2 mM MgOAc) supplemented with a protease inhibitor cocktail (Sigma‐Aldrich, P8340) for 25 min. Following centrifugation at 12 500 g for 15 min at 4°C, the supernatants were incubated with the indicated antibody‐conjugated beads for 1.5 h at 4°C. The beads were then washed four times with lysis buffer, and immunoprecipitated proteins were eluted by boiling and analyzed by immunoblotting.

### 4.14 Subcellular Fractionation

Mitochondrial and mitochondria‐associated ER membrane (MAM) fractions were isolated from mouse liver using an established differential centrifugation protocol [43]. Fresh liver tissue was dissected in ice‐cold Buffer A (30 mM Tris‐HCl, pH 7.4, 0.5% BSA, 0.5 mM EGTA, 75 mM sucrose, and 225 mM mannitol), minced, and homogenized. Nuclei and unbroken cells were removed by repeated centrifugation at 740 g for 5 min at 4°C. The supernatant was then centrifuged at 9000 g for 10 min to obtain crude mitochondria. The resulting supernatant was centrifuged at 20 000 g for 30 min, and the subsequent supernatant at 100 000 g for 1 h (Beckman, 70‐Ti rotor). The final pellet contained the ER‐enriched fraction, whereas the supernatant constituted the cytosolic fraction. The crude mitochondrial pellet was resuspended in Buffer B (as Buffer A without EGTA) and centrifuged at 10 000 g for 10 min. The pellet was then resuspended in Buffer C (5 mM HEPES, pH 7.4, 0.5 mM EGTA, and 250 mM mannitol) and layered onto a Percoll solution (25 mM HEPES, pH 7.4, 1 mM EGTA, 30% Percoll, and 225 mM mannitol). After centrifugation at 95 000 g for 30 min (Beckman, SW40 rotor), the lower (mitochondria) and middle (MAM) bands were collected. Mitochondria were washed by centrifugation at 6300 g, and the MAM fraction was pelleted at 100 000 g for 1 h (70‐Ti rotor, Beckman).

### 4.15 Electron Microscopy

Cells were initially fixed with 2.5% glutaraldehyde in 0.1 M phosphate buffer (1:1 mixture with culture medium) for 3 min at room temperature. The fixative was refreshed once for 30 min. After three 10‑min washes in 0.1 M phosphate buffer, samples were post‑fixed in 1% osmium tetroxide and 0.8% potassium ferrocyanide at room temperature for 30 min. Cells were then rinsed three times with distilled water. Subsequently, samples were dehydrated through a graded ethanol series (30%, 50%, 70%, 85%, 95%, and 100%). Infiltration was performed using pure acetone twice and then using resin: acetone mixtures (1:3 for 30 min; 1:1 for 30 min; 3:1 for 30 min), followed by pure resin under vacuum for 3 h. Polymerization was conducted at 65°C for 24 h. Ultrathin sections were prepared using an ultramicrotome (Leica, EM UC7) and examined with a transmission electron microscope (FEI, Tecnai G2 20 Twin).

For immunoelectron microscopy, samples were prepared following an established protocol [59]. Cells were initially fixed with 4% paraformaldehyde in 0.2 M HEPES buffer (1:1 mixture with culture medium) for 5 min at room temperature. The fixative was refreshed twice: 5 min, then 30 min. After washing with 0.1 M HEPES containing 150 mM glycine, cells were permeabilized with 0.001% saponin in 0.1 M HEPES for 5 min. Cells were then incubated with an anti‐Flag antibody (Proteintech, 20543‐1‐AP; 1:100 dilution), followed by five 5‐min washes with 0.1 M PBS. Subsequently, cells were incubated overnight at 4°C with Nanogold goat anti‐rabbit IgG (Nanoprobes, 2004; 1:200 dilution). After the washes, samples were post‐fixed with 1% glutaraldehyde in 0.2 M HEPES for 30 min and subjected to gold enhancement using GoldEnhance EM Plus (Nanoprobes, 2114) for 2 min. After washing with 1% sodium thiosulfate and distilled water, secondary post‐fixation was performed with 1% osmium tetroxide on ice for 1 h, followed by staining with 2% uranyl acetate for 30 min. Dehydration was performed through a graded ethanol series (30%, 50%, 70%, 85%, 95%, and 100%). Infiltration was performed using resin:ethanol mixtures (1:1 for 2 h; 3:1 for 4 h), followed by pure resin under vacuum for 8 h. Polymerization was conducted at 65°C for 24 h. Ultrathin sections were prepared using an ultramicrotome (Leica, EM UC7) and examined with a transmission electron microscope (FEI, Tecnai G2 Spirit).

### 4.16 Recombinant Protein Production and Pull‐Down Assay

Recombinant proteins were expressed in *Escherichia coli* BL21 cells. Expression was induced with 1 mM isopropyl β‐D‐1‐thiogalactopyranoside, and cultures were incubated at 16°C or 24°C for 16–18 h. Cells were harvested by centrifugation and resuspended in either MBP/GST lysis buffer (20 mM Tris‐HCl, pH 7.5, 200 mM NaCl, 1 mM EDTA, and 1 mM DTT) or His lysis buffer (50 mM Tris‐HCl, pH 8.0, 300 mM NaCl, 4 mM imidazole, and 2 mM β‐mercaptoethanol), supplemented with protease inhibitors. Lysates were sonicated on ice, clarified by centrifugation, and incubated with appropriate affinity resins for 3 h at 4°C. After extensive washing with lysis buffer, bound proteins were eluted using 10 mM maltose (for MBP‐tagged proteins) or 240 mM imidazole (for His‐tagged proteins).

For pull‐down assays, eluted proteins were incubated with GST‐tagged fusion proteins immobilized on Glutathione Sepharose 4B beads (GE Healthcare, 17075601) for 2 h at 4°C. Beads were washed thoroughly and boiled at 100°C for 8 min. Eluates were separated by SDS‐PAGE and analyzed by Coomassie brilliant blue staining and immunoblotting.

### 4.17 Mass Spectrometry Sample Preparation

To identify ATL2 ubiquitination sites under hypoxic conditions, HeLa cells stably expressing 3×Flag‐ATL2 in an ATL2 knockout background were cultured under normoxic or hypoxic conditions. Cells were trypsinized, lysed, and centrifuged to obtain clarified supernatants. These lysates were incubated with anti‐Flag M2 affinity gel (Millipore, A2220) for 3 h at 4°C. After thorough washing, bound proteins were eluted by boiling at 100°C. Immunoprecipitated proteins were separated by SDS–PAGE and visualized with Gel Protein Staining Solution (Meilunbio, MA0399). Gel lanes were excised into slices and subjected to in‐gel tryptic digestion. Digested peptides were analyzed using an Orbitrap Fusion Lumos mass spectrometer (Thermo Fisher Scientific) to map post‐translational modification sites.

### 4.18 Proximity Ligation Assay

For proximity ligation assay (PLA), sample preparation, including primary antibodies incubation, followed the immunofluorescence protocol described above, including fixation, permeabilization, and blocking steps. Subsequent procedures were performed using the Duolink In Situ PLA kit (Sigma‐Aldrich) according to the manufacturer's instructions. Following two PBS washes, PLA probes were applied to samples for 60 min at 37°C. Ligation and amplification reactions were carried out for 30 min and 100 min, respectively. Images were acquired using either a Leica TCS SP8 or a ZEISS LSM 980 confocal microscope equipped with a 63×/1.4 NA oil‐immersion objective. PLA puncta were manually counted using ImageJ.

### 4.19 Statistical Analysis

Statistical analyses were performed using GraphPad Prism 8. Data are presented as means, with error bars representing the indicated measures of variation. Sample sizes (n) and specific statistical tests are provided in the figure legends, while *p*‐values are shown directly on the figures. All quantitative experiments were independently repeated at least three times. For multiple‐group comparisons, a one‐way ANOVA with Tukey's multiple comparisons test or with Dunnett's multiple comparisons test was used. For comparisons between two groups, two‐tailed Student's *t*‐tests were applied to data that fulfilled assumptions of normality and homogeneity of variance; Welch's *t*‐test was used for data with unequal variances. No statistical method was used to predetermine sample size. In experiments with multiple conditions, cells were randomly assigned to each group.

## Author Contributions

Y.C. designed and performed the majority of experiments and drafted the manuscript. P.C. contributed to experimental design and conducted the subcellular fractionation assay. X.P. and Y.C. assisted with molecular biology experiments. P.Z., J.T., and J.C. supervised the project, conceived the study, and provided critical guidance. All authors reviewed, edited, and approved the final version of the manuscript.

## Funding

This work was supported by the National Natural Science Foundation of China (32270740, 32130024, 32470735, 92354306, and 32370732). The study was also supported in part by the Peking‐Tsinghua Center for Life Sciences.

## Conflicts of Interest

The authors declare no conflicts of interest.

## Supporting information




**Supporting File**: advs76972‐sup‐0001‐SuppMat.docx.

## Data Availability

All data generated in this study are available from the corresponding author upon reasonable request. Biological materials, including plasmids, cell lines, and antibodies, will be available on request.
