# Supplementary material for: ATL2 Recruits TRAK1 to Promote Mitochondrial Transport at ER–Mitochondria Contact Sites
Source: Adv Sci (Weinh). 2026 Jul 30:e76972. Online ahead of print. doi: 10.1002/advs.76972 (PMC13423487; doi:10.1002/advs.76972)
Supplement: Supplementary file 1 — Supporting File: advs76972‐sup‐0001‐SuppMat.docx. [file ADVS-9999-e76972-s001.docx]

Supporting Information

ATL2 recruits TRAK1 to promote mitochondrial transport at ER–mitochondria contact sites

Yiru Cheng, Peiyuan Chai, Xiayuhe Pei, Yiwen Chen, Xiaoshuai Huang, Bei Liu, Yiqian Wu, Junlin Teng*, Pengli Zheng*, and Jianguo Chen*


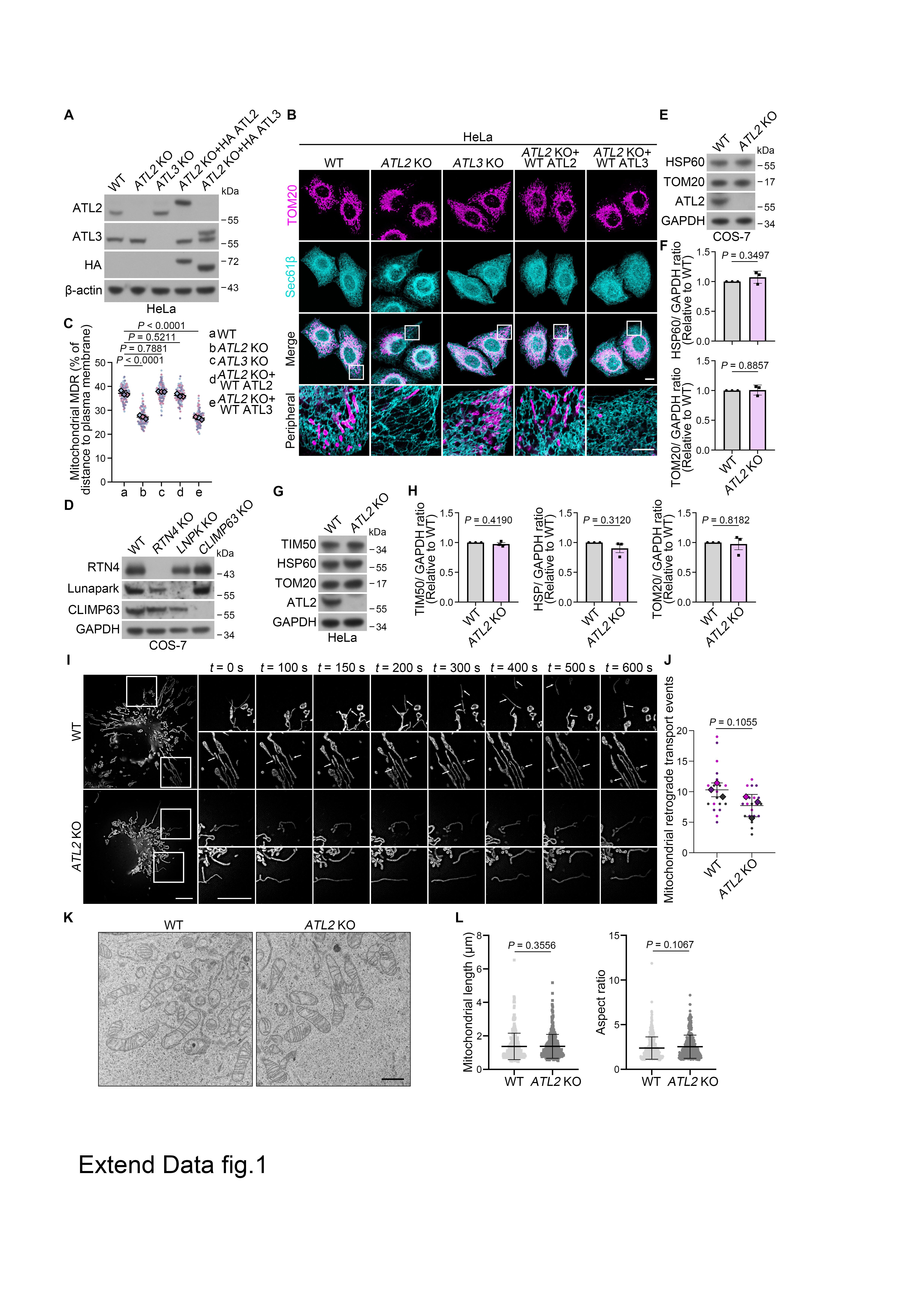
**Supplementary Figures and Legends**

**Figure S1. Mitochondrial transport and distribution in ATL2-deleted cells. Related to Figure 1.**

(**A**) Immunoblot analysis of ATL2 and ATL3 in wild-type (WT), *ATL2* knockout (KO), *ATL3* KO, and *ATL2* KO HeLa cells reconstituted with ATL2 or ATL3. (**B**) Representative images of cells, genotypes as shown in (A), stably expressing 3×mEmerald-Sec61β (cyan, ER marker) and labelled with TOM20 (magenta, mitochondrial marker). Peripheral regions are enlarged at the bottom. Scale bars, 10 μm; 5 µm (inset). (**C**) Mitochondrial mean distribution radius (MDR) in cells as shown in (B). *n* = 107, 105, 112, 106, and 104 cells from three biological replicates. Biological replicates are denoted by color, with individual MDR values depicted as smaller points. Data are presented as mean ± s.d. across biological replicates. (**D**) Immunoblot analysis of RTN4, lunapark, and CLIMP63 in the indicated WT and KO cells. (**E**) Immunoblot analysis of the indicated proteins in WT and *ATL2* KO COS-7 cells. (**F**) Quantification of relative protein levels as shown in (E), with data from three biological replicates presented as mean ± s.e.m. (**G**) Immunoblot analysis of the indicated proteins in WT and *ATL2* KO HeLa cells. (**H**) Quantification of relative protein levels as shown in (G), with data from three biological replicates presented as mean ± s.e.m. (**I**) Time-lapse images of mitochondrial transport in WT and *ATL2* KO COS-7 cells labelled with PK Mito Deep Red (125 nM). Peripheral regions are enlarged on the right. Scale bars, 10 μm; 5 µm (inset). (**J**) Mitochondrial retrograde transport events over 10 min in cells as shown in (I). *n* = 19 and 21 cells from three biological replicates. Biological replicates are denoted by color, with individual transport events depicted as smaller points. Data are presented as mean ± s.d. across biological replicates. (**K**) Representative transmission electron microscopy (TEM) images of mitochondria in WT and *ATL2* KO HeLa cells. Scale bar, 1 μm. (**L**) Quantification of mitochondrial length and aspect ratio as shown in (K). *n* = 368 (WT) and 362 (KO) mitochondria, and data are presented as mean ± s.d. Statistical analyses were performed using ordinary one-way analysis of variance (ANOVA) followed by Tukey’s multiple comparisons test (C); two-tailed unpaired *t*-tests with Welch’s correction (F, H), and without Welch’s correction (J); and two-tailed Mann-Whitney U test (L).


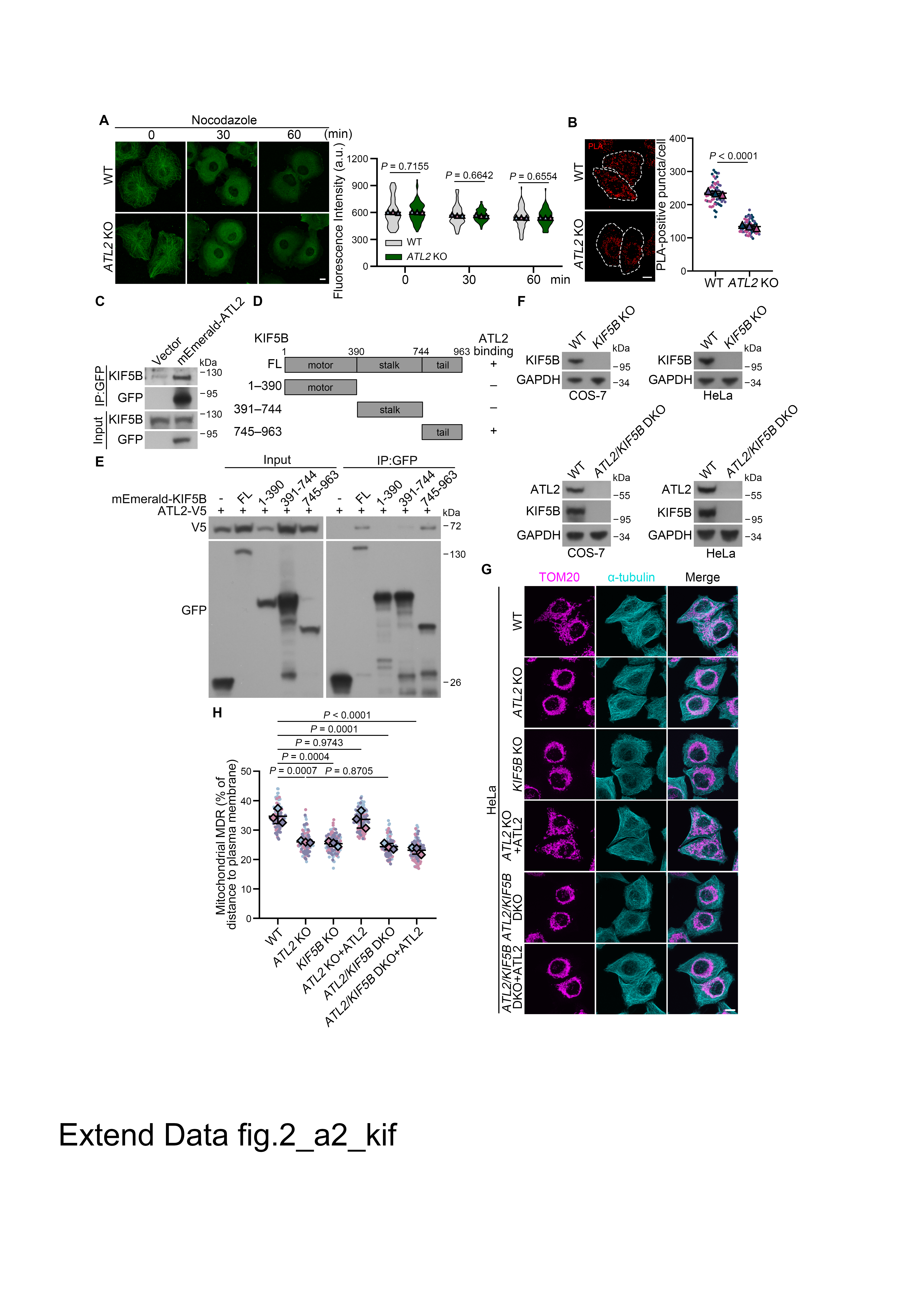
**Figure S2. ATL2 promotes mitochondrial transport in a KIF5B-dependent manner. Related to Figure 2.**

(**A**) Representative images (left) and corresponding quantification of α-tubulin fluorescence intensity (right) in wild-type (WT) and *ATL2* knockout (KO) COS-7 cells treated with nocodazole (10 μM) for the indicated times. Cells were stained with antibodies against α-tubulin (green). Scale bar, 10 μm. *n* = 81, 81, 80, 79, 81 and 79 cells from three biological replicates. Biological replicates are denoted by color. Data are presented as mean ± s.d. across biological replicates. (**B**) Representative images (left) and corresponding quantification (right) of the proximity ligation assay (PLA) detecting endogenous tubulin–TOM20 interactions in WT and *ATL2* KO HeLa cells. Cell outlines are demarcated by white dotted lines. Scale bar, 10 μm. *n* = 60 cells from three biological replicates. Biological replicates are denoted by color, with individual PLA puncta depicted as smaller points. Data are presented as mean ± s.d. across biological replicates. (**C**) HEK293T cells transfected with mEmerald-ATL2 or a control vector were subjected to immunoprecipitation (IP) with anti-GFP nanobody magarose beads. Immunoprecipitates were analysed by immunoblotting using antibodies against GFP and KIF5B. (**D**) Schematic of full-length (FL) KIF5B and the indicated truncated mutants. ATL2 binding ability: +, positive; –, negative. (**E**) HEK293T cells co-transfected with ATL2-V5 and FL mEmerald-KIF5B or the indicated truncated mutants were subjected to immunoprecipitation using anti-GFP nanobody magarose beads. Immunoprecipitates were analysed by immunoblotting using antibodies against GFP and V5. (**F**) Immunoblot analysis of the indicated WT and KO cells. (**G**) Representative images of HeLa cells stained with antibodies against α-tubulin (cyan) and TOM20 (magenta): WT, *ATL2* KO, *KIF5B* KO, *ATL2/KIF5B* double knockout (DKO), *ATL2* KO reconstituted with ATL2, and *ATL2/KIF5B* DKO reconstituted with ATL2. Scale bar, 10 μm. (**H**) Mitochondrial MDR in cells as shown in (G). *n* = 109, 110, 110, 110, 108, and 111 cells from three biological replicates. Biological replicates are denoted by color, with individual MDR values depicted as smaller points. Data are presented as mean ± s.d. across biological replicates. Statistical analyses were performed using two-tailed unpaired *t*-tests (A, B) and ordinary one-way ANOVA followed by Tukey’s multiple comparisons test (H).


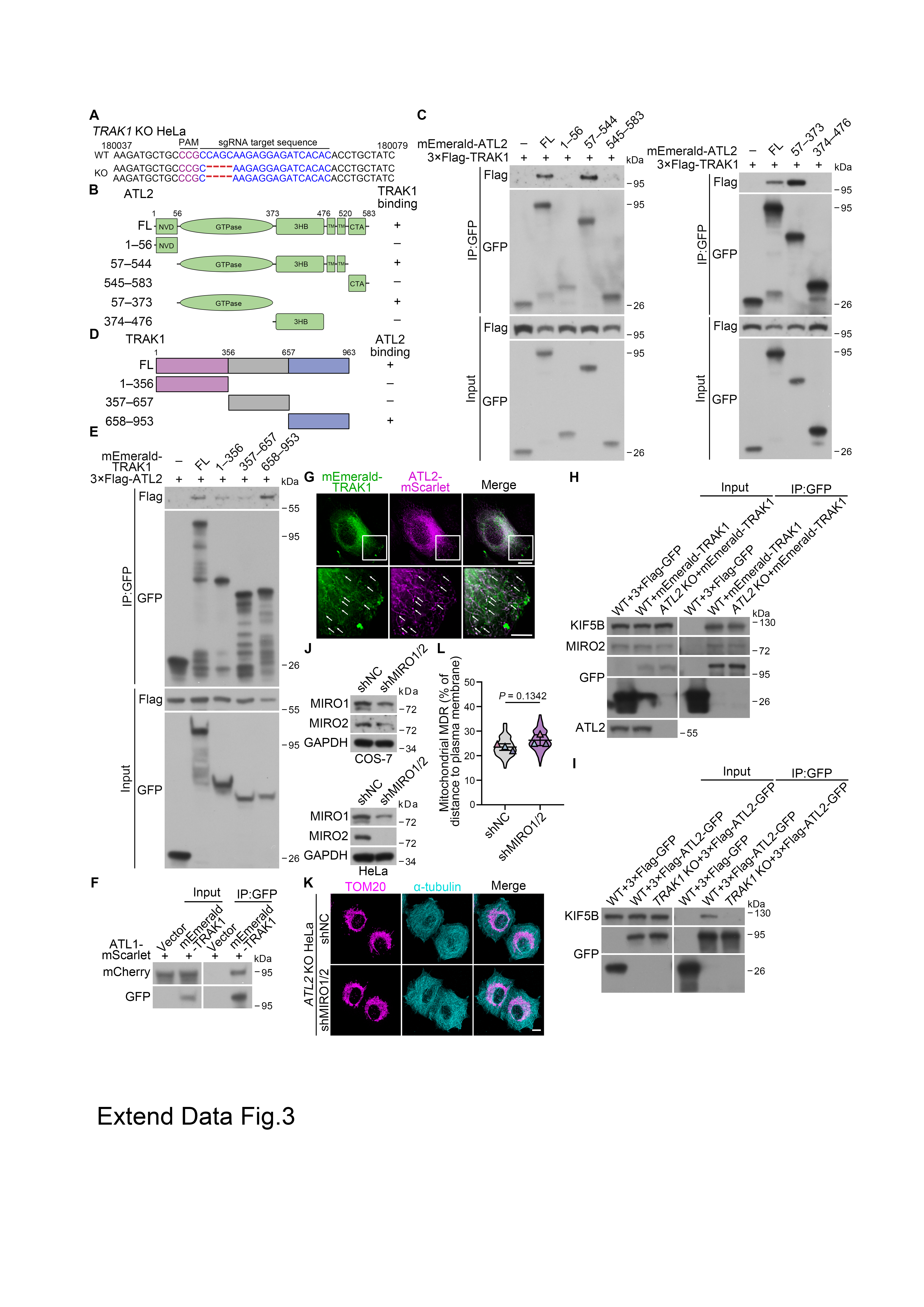
**Figure S3. ATL2 interacts with TRAK1 through its GTPase domain. Related to Figure 3.**

(**A**) Characterization of the genomic sequence in *TRAK1* knockout (KO) HeLa cells. (**B**) Schematic of full-length (FL) ATL2 and the indicated truncated mutants. TRAK1 binding ability: +, positive; –, negative. (**C**) HEK293T cells co-transfected with 3×Flag-TRAK1 and mEmerald-ATL2 FL or the indicated truncated mutants were subjected to immunoprecipitation (IP) using anti-GFP nanobody magarose beads. Immunoprecipitates were analysed by immunoblotting using antibodies against GFP and Flag. (**D**) Schematic of FL TRAK1 and the indicated truncated mutants. ATL2 binding ability: +, positive; –, negative. (**E**) HEK293T cells co-transfected with 3×Flag-ATL2 and mEmerald-TRAK1 (FL or indicated truncated mutants) were subjected to immunoprecipitation using anti-GFP nanobody magarose beads. Immunoprecipitates were analysed by immunoblotting using antibodies against GFP and Flag. (**F**) HEK293T cells co-transfected with ATL1-mScarlet and either mEmerald-TRAK1 or a control vector, were subjected to immunoprecipitation using anti-GFP nanobody magarose beads. Immunoprecipitates were analysed by immunoblotting using antibodies against GFP and mCherry. (**G**) Representative images of HeLa cells co-transfected with mEmerald-TRAK1 and ATL2-mScarlet. Peripheral regions are enlarged at the bottom. White arrows indicate co-localization. Scale bars, 10 μm; 5 µm (inset). (**H**) Lysates from WT or *ATL2* KO HeLa cells stably expressing mEmerald-TRAK1, or from WT cells expressing 3×Flag-GFP, were incubated with anti-GFP nanobody magarose beads. Immunoprecipitates were analysed by immunoblotting using antibodies against GFP, KIF5B, MIRO2, and ATL2. (**I**) Lysates from WT or *TRAK1* KO HeLa cells stably expressing 3×Flag-ATL2, or WT cells expressing 3×Flag-GFP, were incubated with anti-GFP nanobody magarose beads. Immunoprecipitates were analysed by immunoblotting using antibodies against GFP and KIF5B. (**J**) Immunoblot analysis of *ATL2* KO cells with or without additional knockdown of MIRO1/2. (**K**) Representative images of *ATL2* KO HeLa cells with or without additional depletion of MIRO1/2 stained with antibodies against α-tubulin (cyan) and TOM20 (magenta). Scale bar, 10 μm. (**L**) Mitochondrial MDR in cells as shown in (K). *n* = 109 and 110 cells from three biological replicates. Biological replicates are denoted by color. Data are presented as mean ± s.d. across biological replicates. Statistical analyses were performed using two-tailed unpaired *t*-tests.


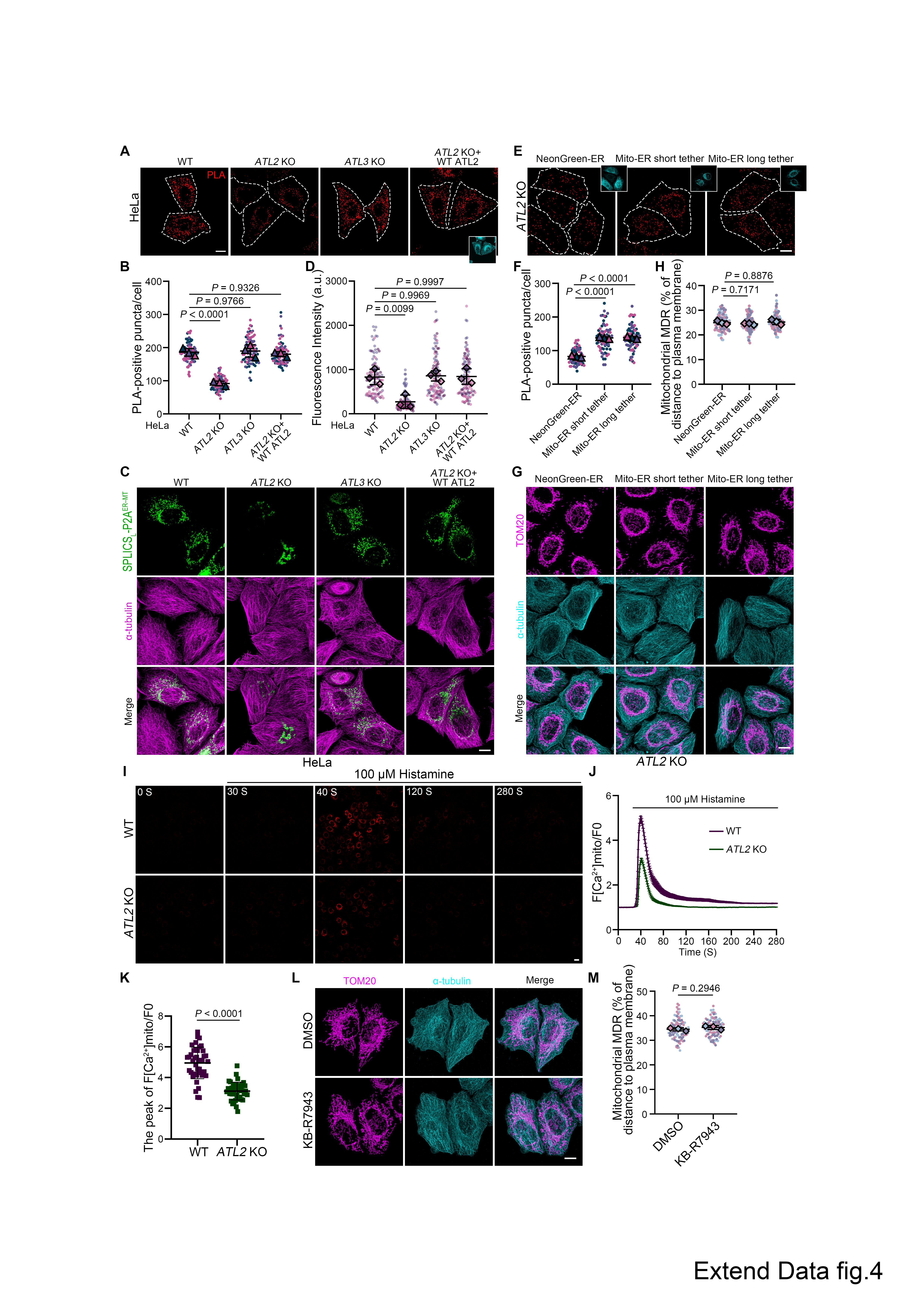
**Figure S4. Loss of ATL2 disrupts ER–mitochondria contact sites. Related to Figure 4.**

(**A**) PLA detecting the endogenous interaction between TOM20 and calnexin in wild-type (WT), *ATL2* knockout (KO), *ATL3* KO, and *ATL2* KO HeLa cells reconstituted with ATL2. Cell outlines (white dotted lines) and expressed ATL2 (cyan) are indicated. Scale bar, 10 μm. (**B**) Quantification of PLA puncta per cell as shown in (A). *n* = 93, 93, 92, and 94 cells from three biological replicates. Biological replicates are denoted by color, with individual PLA puncta depicted as smaller points. Data are presented as mean ± s.d. across biological replicates. (**C**) Representative images of WT, *ATL2* KO, *ATL3* KO, and *ATL2* KO HeLa cells reconstituted with ATL2, expressing SPLICS_L_-P2A^ER-MT^ (green) and stained with antibodies against α-tubulin (magenta). Scale bar, 10 μm. (**D**) Quantification of green fluorescence intensity per cell area as shown in (C). *n* = 108, 102, 108, and 110 cells from three biological replicates. Biological replicates are denoted by color, with individual data points depicted as smaller points. Data are presented as mean ± s.d. across biological replicates. (**E**) PLA detecting the endogenous interaction between TOM20 and calnexin in *ATL2* KO HeLa cells expressing synthetic mitochondria–ER tether constructs (cyan). Cell outlines are demarcated by white dotted lines. Scale bar, 10 μm. (**F**) Quantification of PLA puncta per cell as shown in (E). *n* = 66, 68, and 68 cells from three biological replicates. Biological replicates are denoted by color, with individual PLA puncta depicted as smaller points. Data are presented as mean ± s.d. across biological replicates. (**G**) Representative images of *ATL2* KO HeLa cells expressing synthetic mitochondria–ER tether constructs stained with antibodies against α-tubulin (cyan) and TOM20 (magenta). Scale bar, 10 μm. (**H**) Mitochondrial MDR in cells as shown in (G). *n* = 90, 92, and 89 cells from three biological replicates. Biological replicates are denoted by color, with individual MDR values depicted as smaller points. Data are presented as mean ± s.d. across biological replicates. (**I**) Representative images of WT and *ATL2* KO HeLa cells expressed mito-R-GECO, a mitochondrial Ca^2+^ indicator. Histamine (100 μM) was added at 30 s of imaging Scale bars, 20 μm. (**J**) Quantification of fluorescence intensity of the Ca^2+^ indicator over time. Traces represent fluorescence intensity normalized to the value at 0 s (F/F₀). *n* = 41 cells from three biological replicates. (**K**) Quantification of the Ca^2+^ indicator peak intensity in (J). (**L**) Representative images of WT HeLa treated with DMSO or KB‑R7943 (10 μM) and stained with antibodies against α-tubulin (cyan) and TOM20 (magenta). Scale bar, 10 μm. (**M**) Mitochondrial MDR in cells as shown in (L). *n* = 105 cells from three biological replicates. Biological replicates are denoted by color, with individual MDR values depicted as smaller points. Data are presented as mean ± s.d. across biological replicates. Statistical analyses were performed using ordinary one-way ANOVA followed by Tukey’s multiple comparisons test (B, D, F and H); two-tailed unpaired *t*-tests with Welch’s correction (K), and without Welch’s correction (M).


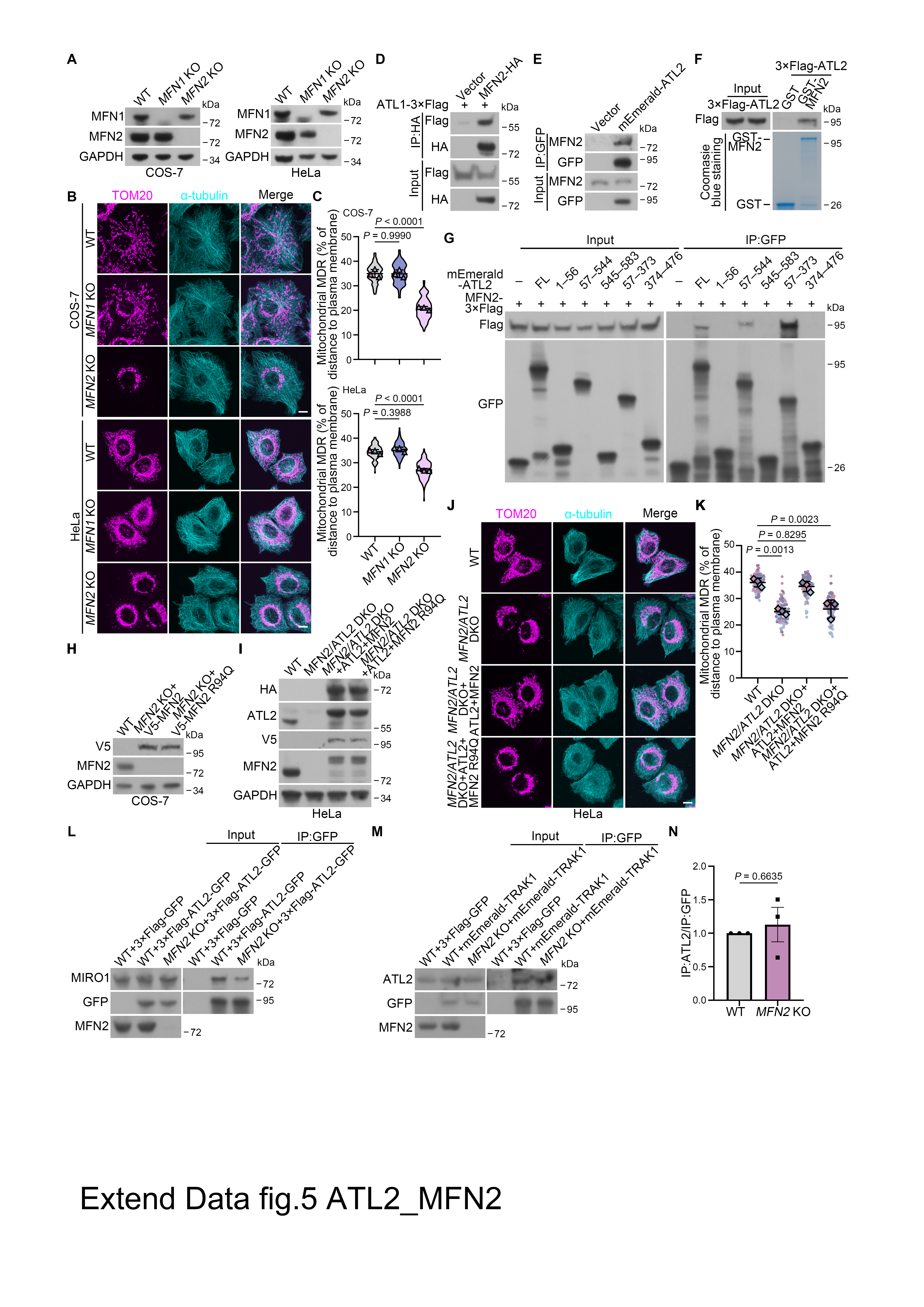
**Figure S5. ATL2 interacts with MFN2. Related to Figure 5.**

(**A**) Immunoblot analysis of MFN1 and MFN2 in the indicated wild-type (WT) and knockout (KO) cells. (**B**) Representative images of WT, *MFN1* KO, and *MFN2* KO cells stained with antibodies against α-tubulin (cyan) and TOM20 (magenta). Scale bar, 10 μm. (**C**) Mitochondrial MDR in cells as shown in (B). *n* = 106, 106, 106, 107, 108, and 106 cells from three biological replicates. Biological replicates are denoted by color. Data are presented as mean ± s.d. across biological replicates. (**D**) HEK293T cells co-transfected with ATL1-3×Flag and either MFN2-HA or a control vector were subjected to immunoprecipitation (IP) using anti-HA nanobody magarose beads. Immunoprecipitates were analysed by immunoblotting using antibodies against HA and Flag. (**E**) HEK293T cells transfected with mEmerald-ATL2 or a control vector were subjected to immunoprecipitation using anti-GFP nanobody magarose beads. Immunoprecipitates were analysed by immunoblotting using antibodies against GFP and MFN2. (**F**) Lysates from *ATL2* KO HeLa cells expressing 3×Flag-ATL2 were subjected to affinity isolation with immobilized GST or GST-MFN2. Immunoblots and Coomassie blue-stained gels are shown, probed with an antibody against Flag. (**G**) HEK293T cells co-transfected with MFN2-3×Flag together with full-length (FL) mEmerald-ATL2 or the indicated truncated mutants were subjected to immunoprecipitation with anti-GFP nanobody magarose beads. Immunoprecipitates were analysed by immunoblotting using antibodies against GFP and Flag. (**H**) Immunoblot analysis of WT COS-7 cells, and *MFN2* KO COS-7 cells reconstituted with V5-WT MFN2 or V5-MFN2 R94Q. (**I**) Immunoblot analysis of WT HeLa cells, *MFN2/ATL2* double-knockout (DKO) HeLa cells, and DKO cells reconstituted with HA-ATL2 together with either V5-WT MFN2 or V5-MFN2 R94Q. (**J**) Representative images of WT HeLa cells, *MFN2/ATL2* DKO HeLa cells, and DKO cells reconstituted with ATL2 together with either WT MFN2 or the MFN2 R94Q mutant, stained with antibodies against α-tubulin (cyan) and TOM20 (magenta). Scale bar, 10 μm. (**K**) Mitochondrial MDR in cells as shown in (J). *n* = 110, 108, 110, and 108 cells from three biological replicates. Biological replicates are denoted by color, with individual MDR values depicted as smaller points. Data are presented as mean ± s.d. across biological replicates. (**L**) Lysates from WT or *MFN2* KO HeLa cells stably expressing 3×Flag-ATL2-GFP, or WT cells expressing 3×Flag-GFP, were incubated with anti-GFP nanobody magarose beads. Immunoprecipitates were analysed by immunoblotting using antibodies against GFP, MIRO1, and MFN2. (**M**) Lysates from WT or *MFN2* KO HeLa cells stably expressing mEmerald-TRAK1, or WT cells expressing 3×Flag-GFP, were incubated with anti-GFP nanobody magarose beads. Immunoprecipitates were analysed by immunoblotting using antibodies against GFP, ATL2, and MFN2. (**N**) Quantification of relative intensity as shown in (M), with data from three biological replicates presented as mean ± s.e.m. Statistical analyses were performed using ordinary one-way ANOVA followed by Tukey’s multiple comparisons test (C and K) and two-tailed unpaired *t*-tests with Welch’s correction (N).


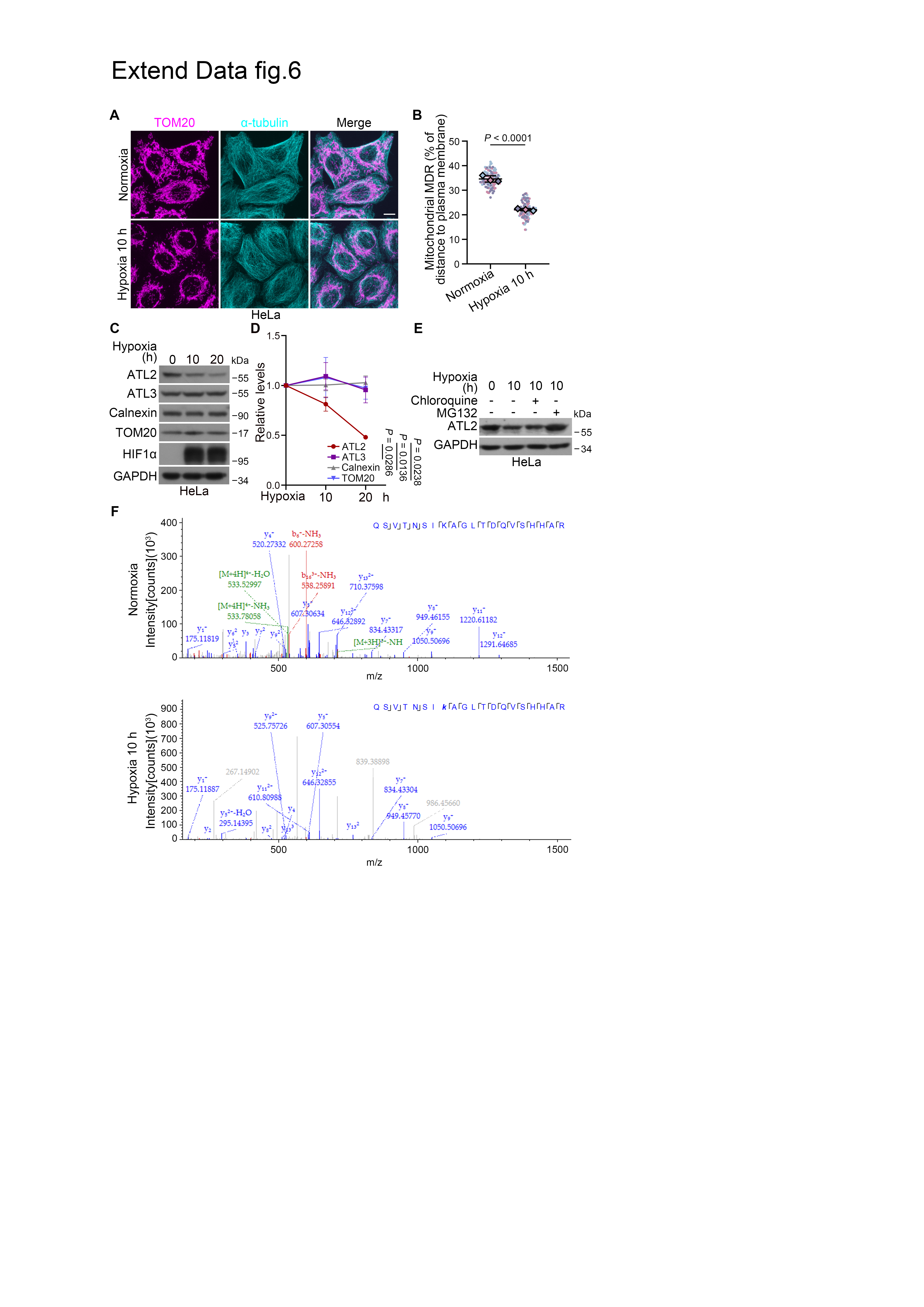
**Figure S6. Loss of ATL2 under hypoxia causes perinuclear mitochondrial clustering and involves K567 ubiquitination. Related to Figure 6.**

(**A**) Representative images of HeLa cells exposed to 1% O_2_ for the indicated times and stained with antibodies against α-tubulin (cyan) and TOM20 (magenta). Scale bar, 10 μm. (**B**) Mitochondrial MDR in cells as shown in (A). *n* = 108 and 107 cells from three biological replicates. Biological replicates are denoted by color, with individual MDR values depicted as smaller points. Data are presented as mean ± s.d. across biological replicates. (**C**) Immunoblot analysis of ATL2, ATL3, calnexin, and TOM20 in HeLa cells exposed to 1% O_2_ for the indicated times. (**D**) Quantification of relative protein levels following 20 h of hypoxia as shown in (C), with data from three biological replicates presented as mean ± s.e.m. (**E**) Immunoblot analysis of ATL2 in HeLa cells exposed to 1% O_2_ for the indicated times with MG132 (10 μM) or chloroquine (10 μM). (**F**) Mass spectrometry analysis of ubiquitination at K567 of Flag-tagged ATL2 in HeLa cells exposed to1% O₂ for the indicated times. Statistical analyses were performed using two-tailed unpaired *t*-tests (B) and ordinary one-way ANOVA followed by Tukey’s multiple comparisons test (D).


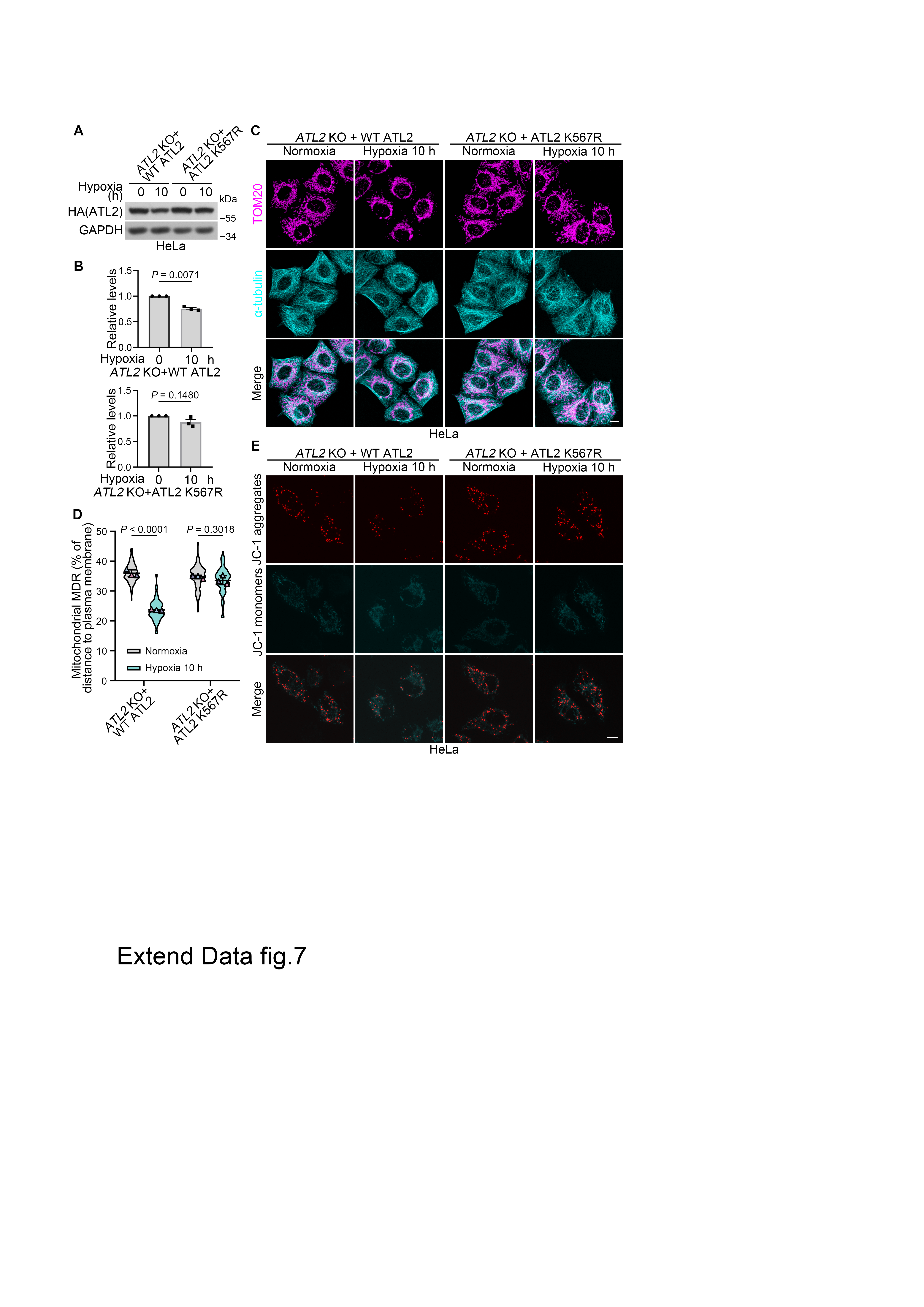
**Figure S7. The ATL2 K567R mutant rescues mitochondrial distribution and suppresses ROS elevation under hypoxia. Related to Figure 6.**

(**A**) Immunoblot analysis of HA protein levels in *ATL2* knockout (KO) HeLa cells stably expressing HA-WT ATL2 or HA-ATL2 K567R, exposed to 1% O_2_ for the indicated times. (**B**) Quantification of relative protein levels as shown in (A), with data from three biological replicates presented as mean ± s.e.m. (**C**) Representative images of *ATL2* KO HeLa cells stably expressing WT ATL2 or the ATL2 K567R mutant exposed to 1% O_2_ for 0 or 10 h stained with antibodies against α-tubulin (cyan) and TOM20 (magenta). Scale bar, 10 μm. (**D**) Mitochondrial MDR in cells as shown in (C). *n* = 106, 108, 102, and 102 cells from three biological replicates. Biological replicates are denoted by color. Data are presented as mean ± s.d. across biological replicates. (**E**) Representative images of *ATL2* KO HeLa cells stably expressing WT ATL2 or the ATL2 K567R mutant, exposed to 1% O_2_ for 0 or 10 h, stained with JC-1 (5 µg/mL). Scale bar, 10 μm. Statistical analyses were performed using two-tailed unpaired *t*-tests with (B) or without (D) Welch’s correction.
